# Predictors of self-reported adherence to COVID-19 guidelines. A longitudinal observational study of 51,600 UK adults

**DOI:** 10.1016/j.lanepe.2021.100061

**Published:** 2021-05

**Authors:** Liam Wright, Andrew Steptoe, Daisy Fancourt

**Affiliations:** a Department of Behavioural Science and Health, University College London, 1-19 Torrington Place, London WC1E 7HB, United Kingdom; b Department of Epidemiology and Public Health, University College London, 1-19 Torrington Place, London WC1E 7HB, United Kingdom

## Abstract

**Background:**

In the absence of a vaccine, governments have focused on social distancing, self-isolation, and increased hygiene procedures to reduce the transmission of SARS-CoV-2 (COVID-19). Compliance with these measures requires voluntary cooperation from citizens. Yet, compliance is not complete. Existing research on the predictors of compliance is almost exclusively based on cross-sectional data, raising the possibility of reverse causality and confounding.

**Methods:**

Using data from the UCL COVID-19 Social Study, a large weekly online panel of UK adults from first three months of lockdown in the UK (n = 51,600), we tested whether within-person changes in confidence in government, mental wellbeing, social experiences and awareness of COVID-19 were longitudinally related to self-reported compliance levels with guidelines from authorities using random intercept cross-lagged panel models.

**Findings:**

We found evidence of a small longitudinal association between increased confidence in government to tackle the pandemic and higher self-reported compliance, but little evidence that factors such as mental health and wellbeing, worries about future adversities, and social isolation and loneliness were related to later compliance. We found higher self-reported compliance was longitudinally related to higher depressive symptoms. We found that low compliance was related to lower leisure engagement, providing care, and working outside the home.

**Interpretation:**

Our results suggest that to effectively manage the pandemic, governments should ensure that confidence is maintained.

**Funding:**

Nuffield Foundation, Wellcome Trust and the MARCH Mental Health Network. MARCH is funded by the Cross-Disciplinary Mental Health Network Plus initiative supported by UK Research and Innovation.

**Research in context**

**Evidence before this study**

We undertook searches of Google Scholar for published and pre-print scientific articles related to compliance with COVID-19 measures, using the keywords “COVID-19″, “compliance”, “comply”, “adherence”, “adhere”, “follow”, “rules”, “guidelines”, and “preventative”. We followed these searches with forward and backward citation searching. A sizeable literature was identified, but much of this literature is based on cross-sectional data and focuses on fixed person characteristics, rather than modifiable factors. This raises the possibility that much of the literature is beset by issues of reverse causality and confounding.

**Added value of this study**

Using a large longitudinal sample of UK adults across three months of lockdown in the UK, we were able to test whether within-person changes in factors related to confidence in government, mental wellbeing, social experiences and awareness of COVID-19 were longitudinally related to self-reported compliance levels. Our statistical approach – random intercept cross-lagged panel models – allowed us to account for time-invariant between-person heterogeneity and to test for reverse causality. We found that increased confidence in the UK government to tackle the pandemic was longitudinally related to higher compliance, but little evidence that the other studied factors were related to later changes in compliance. We did find large between-person correlations between compliance and several of the studied factors, however, suggesting that cross-sectional associations are confounded.

**Implications of all the available evidence**

Our results suggest that to effectively manage the pandemic, governments should ensure that confidence is maintained. The results also suggest that cross-sectional studies of compliance behaviour should be interpreted with caution.

Alt-text: Unlabelled box

The pandemic of SARS-CoV-2 (COVID-19) has had wide ranging impacts on societies across the globe. In the absence of a vaccine, governments have implemented a range of measures to tackle the pandemic, generally focused on reducing transmission of the virus through: isolating those with diagnosed or suspected COVID-19, increasing ‘social distancing’ (e.g. working from home, restricting non-essential travel and limiting groups gathering in public venues), and enhancing hygiene procedures (such as the wearing of face masks). Existing evidence suggest the measures could have large impacts on infection rates and, subsequently, on reducing overall mortality [1]. However, each of these measures requires citizens to make changes to their usual behaviour, sometimes at considerable personal cost. Though some measures have the force of law, in democratic societies unwilling to exercise authoritarian power, compliance requires voluntary cooperation. Yet, ensuring high levels of compliance has been a challenge. To manage the pandemic effectively, it is vital that we understand the factors that drive compliance; especially those factors that could be modifiable.

The role of the factors in determining compliance may be explicated using models of (health) behaviour, such as the COM-B model [2], which posits that behaviour reflects (objective and subjective) capability, opportunity for action, and motivation (i.e. perceived costs and benefits). There is a moderate literature from previous epidemics, such as the 2009 H1N1 swine flu and the 2003 severe acute respiratory syndrome (SARS), on factors that influence compliance with social distancing, hygiene, and quarantine rules [3]. This literature highlights several factors including confidence in institutions (which can affect motivations), social experiences (which can affect capabilities, opportunities, and motivations), mental health and wellbeing, and knowledge of the virus (which can affect capability and motivations). Each of these is discussed below.

With regards to *confidence in institutions*, trust and confidence in government can increase motivations to comply by assuring citizens that guidelines are necessary and effective [4]. During the Ebola epidemic in Liberia, individuals with greater trust in government were more likely to comply with mandated social distancing [5], whilst during COVID-19, cross-sectional studies have shown an association between trust in governmental figures and compliance with protective measures [6]. Reductions in geographical mobility – an indicator of adherence with lockdown and social distancing regulations – were also greater in regions of Europe with higher pre-pandemic levels of trust in politicians [7]. However, a limitation of these studies is their focus on the early stages of the pandemic: a relationship was found between trust and compliance only at the start of the H1N1 pandemic in the Netherlands [8]. Moreover, confidence may be a “double-edged sword” if citizens feel that success is assured regardless of their own actions [9].

Compliance may additionally be related to *mental health and wellbeing*, although empirical results and influential models of the relationship between positive affect and prosocial and health-promoting behaviour make conflicting predictions [10]. On the one hand, depression and anxiety are related to lower extraversion, sociability [11] and increased risk aversion [12], which could increase motivations to comply. Anxiety has been shown to be higher during the COVID-19 pandemic, partly driven by specific fears about catching the virus, with data suggesting both these state worries and trait anxiety levels may encourage compliance [3,13]. On the other hand, depression has been associated with lower self-efficacy and lower altruism [14,15] and is linked to non-compliance with medical treatments, more generally [16]. Further, longitudinal research during COVID-19 has suggested that lower life satisfaction is related to lower compliance [10], though lagged life satisfaction had the opposite association. As both depression and wellbeing have been adversely affected during the COVID-19 pandemic, it would therefore be concerning if these factors are indeed related to lower compliance. Studies using cross-sectional data from Japan, China and the UK during the COVID-19 pandemic are not consistent in their findings, so the relationship between mental health, wellbeing and compliance is not well understood at present [13,17,18]. This lack of consistency may be because the relationship between mental health, wellbeing and compliance is likely bidirectional, as compliance with quarantine and social distancing may itself be a cause of poor mental health [19]. Studies that seek to disentangle the direction of this relationship are particularly needed.

*Knowledge and exposure to information about COVID-19* may increase motivations to comply by increasing awareness of the risks of catching COVID-19. Further, such information may increase capabilities by offering strategies for compliance. However, it may also widen exposure to violations of social norms, which may discourage pro-social motivations to comply with guidelines [20], and to information that could induce fatalism [21]. Multiple cross-sectional studies have already shown that knowledge about COVID-19 is related to greater self-reported compliance with preventative measures [22,23]. However, these associations could be explained by individuals with greater willingness to comply also being more likely to seek out information about COVID-19. Notably, there is experimental evidence that providing higher expert estimates of infectiousness can *reduce* reported willingness to follow social distancing measures [21], suggesting that whether improved knowledge increases compliance depends on pre-existing beliefs. Individuals who seek out information may also be more trusting in general.

A fourth group of factors that may affect compliance are *social experiences*. An important question is the extent to which social isolation impacts compliance, given that compliance with social distancing measures affect loneliness and interpersonal contact [19]. People may seek to relieve loneliness by breaking lockdown and social distancing rules. Loneliness is also related to poorer health practices and health-promoting behaviours, with behavioural disengagement in the face of stressors, and with lower perceptions of control [24]. However, direct empirical evidence from the COVID-19 pandemic is limited with only cross-sectional evidence that loneliness is related to lower compliance [25].

Finally, there is reason to believe that *time-use* during quarantine could be related to compliance, likely in a bi-directional way. Some activities may make compliance more pleasant, reducing boredom, improving wellbeing, and incentivising people to stay at home. Performing specific activities, such as working outside the home or caring for friends or relatives, may also pose a challenge for compliance, either due to the inevitable consequence of individuals’ careers (such as essential workers being unable to stay at home during strict lockdowns) or due to economic necessity. Therefore, investigating how compliance co-varies with time use could help to identify groups who need more support to comply with guidelines.

Overall, then, there is preliminary evidence to suggest that a range of factors could be related to compliance during COVID-19. However, to date, most of the literature on compliance during COVID-19 has used cross-sectional data, which raises the possibility that associations are explained by reverse causality or unobserved confounding. Further, there has been little research directly comparing the size of association between different predictive factors and compliance. Such research is important for public health professionals and policy makers deciding what sorts of messaging and interventions are needed to maintain high adherence to try and control the spread of the virus. Therefore, in this paper we used data from a weekly panel of 51,600 adults across twelve weeks of lockdown in the UK (01 April – 22 June) to explore which factors out of a wide range drawn from the literature cited above were associated with self-reported adherence to government guidelines to tackle COVID-19.

## 1 Methods

### 1.1 Participants

We used data from the COVID-19 Social Study; a large panel study of the psychological and social experiences of over 75,000 adults (aged 18+) in the UK during the COVID-19 pandemic. The study commenced on 21 March 2020 and involves online weekly data collection for the duration of the pandemic in the UK. The study is not random and therefore is not representative of the UK population, but does contain a well-stratified sample. The sample was recruited using three primary approaches. First, snowballing was used, including promoting the study through existing networks and mailing lists (including large databases of adults who had previously consented to be involved in health research across the UK), print and digital media coverage, and social media. Second, more targeted recruitment was undertaken focusing on (i) individuals from a low-income background, (ii) individuals with no or few educational qualifications, and (iii) individuals who were unemployed. Third, the study was promoted via partnerships with third sector organisations to vulnerable groups, including adults with pre-existing mental health conditions, older adults, carers, and people experiencing domestic violence or abuse. Participants were followed by email. Participants received an invitation to the next wave of data collection 7 days following their last completion, with reminders 24 and 48 hours following their initial weekly invitation. If they did not complete the survey following these reminders, the stopped receiving future surveys, but the link from their last reminder remained live so they could return to the study a few days late if they chose. The study was approved by the UCL Research Ethics Committee [12,467/005] and all participants gave informed consent. The study protocol and user guide (which includes full details on recruitment, retention, data cleaning, weighting and sample demographics) are available at https://github.com/UCL-BSH/CSSUserGuide.

**Fig. 1:**
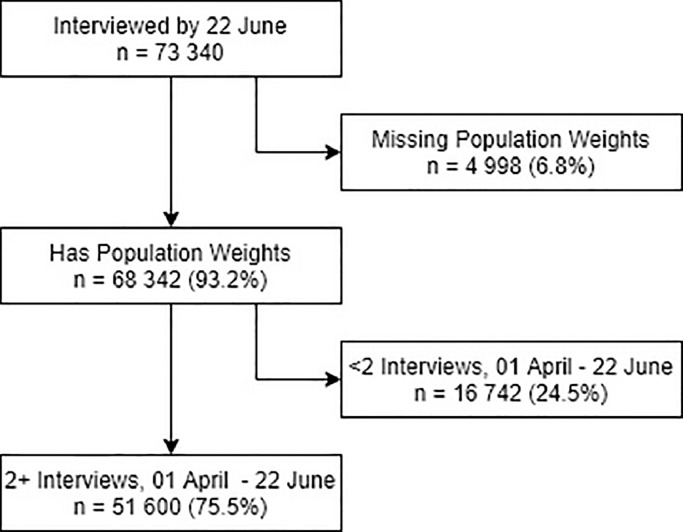
Study flow diagram.

Lockdown began in the UK on 23 March 2020. For these analyses, we focused on participants with 2+ data collections between 01 April – 22 June with key demographic data which we use to construct survey weights (*n* = 54,155, 70.4% of individuals responding by 22 June). A study flow diagram is shown in Fig. 1. Recruitment into the study was ongoing across this period. We used complete case analysis as there was only a small amount of item missingness in the study (minimum sample size 47,380).

### 1.2 Measures

#### 1.2.1 Compliance with guidelines

*Compliance with guidelines* was measured weekly using a single-item measure: “Are you following the recommendations from authorities to prevent spread of Covid-19?” The item was measured on a seven-point Likert scale, 1 = Not at all – 7 = Very much so, and analysed as a continuous variable.

#### 1.2.2 Predictors of compliance

Full details of our measures are provided in Supplementary Material. For *mental health and wellbeing*, we measured depression (9-Item Patient Health Questionnaire), anxiety (7-Item Generalised Anxiety Disorder), meaning in life and happiness (Office for National Statistics wellbeing measures), sleep quality, and number of life stressors. For *social experiences*, we measured the number of days over the past week that the participant had not left the house or garden (home isolation), had face-to-face contact for 15 min or more (face-to-face isolation), and had a phone or video call contact with someone for 15 min or more (phone isolation), and their loneliness levels (3-item UCLA-3 Loneliness scale). For *confidence in institutions*, we measured confidence in devolved government, the health system, and access to essentials (such as food, water, electricity etc.). Participants in devolved nations were asked to answer specifically on devolved governments and health systems. *COVID-19 awareness* was measured with questions on self-rated knowledge about COVID-19 and time spent reading, watching the news, or listening to radio broadcasts about COVID-19 and time spend tweeting, blogging, or posting content online about COVID-19. Finally, *time use* was measured by asking participants how much time they spent on different activities including work, childcare, home chores, and leisure (see Table 1).

**Table 1:** Questionnaire items on time use.

Activity	Questionnaire Items
*Remote working*	- Phoning or video talking with colleagues whilst working remotely - Undertaking other work remotely
*Working outside house*	- Going to work outside of the house (e.g. to the office)
*Caring for friends or relatives*	- Caring for a friend or relative
*Childcare*	- Caring for children (e.g. bathing, feeding, doing homework with etc.) - Playing with children (e.g. general play or board games or card games)
*Volunteer work*	- Volunteering
*Household chores*	- Household chores (cooking, cleaning, ironing, tidying, online shopping etc.)
*Exercise*	- Going out for a walk or other gentle physical activity - Going out for moderate or high intensity activity (e.g. running, cycling or swimming)
*Community group engagement*	- Going out of the house to engage in a community group
*Arts and crafts*	- Engaging in a home-based arts or crafts activity (e.g. painting, creative writing, sewing, playing music, etc.) - Engaging in a digital arts activity (e.g. streaming a concert, virtual tour of a museum etc.) - Doing DIY, woodwork, metal work, model making or similar
*Broader leisure*	- Playing video or computer games alone, or with adults or children - Watching TV, films, Netflix etc. (NOT for information on Covid-19) - Browsing the internet (NOT for information on Covid-19) - Procrastinating or not doing anything in particular

### 1.3 Analysis

We analysed time-use and non-time-use measures separately. To analyse the longitudinal relationship between the non-time-use measures and self-reported compliance, we estimated Random Intercept-Cross Lagged Panel Models (RI-CLPM) [26]. The RI-CLPM is specified in a structural equation modelling framework. It adds correlated latent random intercept factors to the standard cross lagged panel model. Lagged paths are modelled between observation-level residuals once person-specific means are accounted for (see Supplementary Fig. S1). This allows for the separation of within-person variation from between-person variation when estimating cross-lagged effects. Accordingly, it can be used to answer the question whether *changes* in one variable are followed by changes in the other (and vice versa), rather than confounding longitudinal with cross-sectional variation, which the standard CLPM does [26].

We estimated a separate RI-CLPM for each non-time-use measure defined above, using the full sample and, as a sensitivity analysis, also stratifying by gender as prior work shows some differences in compliance between males and females [27]. As individuals vary according to number of follow-ups, we used the full-information maximum likelihood (FIML) estimator in order to use data from all participants, rather than from a balanced panel. We restricted path coefficients to be equal across time, given panel imbalance and participants being able to enter the survey on different dates. We included linear time trends in the model to account for strong time trends in some of the variables in this analysis (Supplementary Information Figs. S2 and S3). Models did not always converge with more complex adjustments for time (e.g., date fixed effects), so as a further sensitivity analysis we ran fixed effects models with different adjustments for time trends to test how results differed. The RI-CLPM models used data from up to 11 waves for each participant. Models were fit using the lavaan R package version 0.6–6 [28] with the nlminb optimiser. Further details on the RI-CLPM methods and fit statistics are in the Supplementary Material.

To analyse the association between the time-use measures and self-reported compliance, we ran standard fixed effects regression models. These models included time use and compliance measures from the same wave, rather than lagged effects, as we are interested in the activities that individuals carry out when they are not complying. We estimated models for each time-use measure separately and a further model including all time-use measures together. To account for time trends in the data, we added date fixed effects into each model.

As we analysed multiple variables in this study, we report Bonferroni corrected confidence intervals and standardised effect sizes (standardised using within-person standard deviations). We included survey weights in models to account for the non-representativeness of the initial sample. The weighted data were matched to population statistics across the following characteristics: age, gender, ethnicity, education, and country of living. Population statistics were taken from the ONS's Annual Population Survey [29].

### 1.4 Role of the funding source

The funders had no final role in the study design; in the collection, analysis and interpretation of data; in the writing of the report; or in the decision to submit the paper for publication. All researchers listed as authors are independent from the funders and all final decisions about the research were taken by the investigators and were unrestricted.

## 2 Results

### 2.1 Descriptive statistics

Descriptive statistics on the sample characteristics are displayed in Table 2. Further descriptive data on study measures, including breakdowns by waves completed, are shown in Supplementary Tables S2-S4. There were 51,600 eligible participants. Participants completed 7.3 waves, on average, with 25% of the sample completing 11 or more waves of data collections over the analysis period. Note, as participants entered the survey at different points, fewer data collections does not necessarily imply dropout from the survey. Younger participants and those with lower compliance levels were more likely to complete fewer waves of data collection (Supplementary Table S3 and Fig. S4).

**Table 2:** Sample characteristics.

		Unweighted		Weighted	
	Variable	Individuals (%)	Observations (%)	Individuals (%)	Observations (%)
Gender	Male	12,737 (24.68%)	95,062 (25.14%)	24,915.95 (48.29%)	185,664.11 (49.11%)
	Female	38,863 (75.32%)	283,001 (74.86%)	26,684.05 (51.71%)	192,398.89 (50.89%)
Country of residence	England	41,604 (80.63%)	304,150 (80.45%)	43,463.98 (84.23%)	317,651.81 (84.02%)
	Wales	6164 (11.95%)	45,895 (12.14%)	2889.89 (5.6%)	22,086.02 (5.84%)
	Scotland	3296 (6.39%)	24,286 (6.42%)	4121.02 (7.99%)	30,711.93 (8.12%)
	Northern Ireland	536 (1.04%)	3732 (0.99%)	1125.11 (2.18%)	7613.24 (2.01%)
Age group	18–29	4081 (7.91%)	24,220 (6.41%)	7184.89 (13.92%)	41,044.62 (10.86%)
	30–45	14,390 (27.89%)	94,788 (25.07%)	12,039.87 (23.33%)	78,203.14 (20.69%)
	46–59	16,596 (32.16%)	121,747 (32.2%)	13,620.67 (26.4%)	100,212.79 (26.51%)
	60+	16,533 (32.04%)	137,308 (36.32%)	18,754.57 (36.35%)	158,602.45 (41.95%)
Highest qualification	GCSE or below	7005 (13.58%)	51,028 (13.5%)	16,086.29 (31.17%)	118,977.30 (31.47%)
	A-levels or equivalent	9019 (17.48%)	64,954 (17.18%)	17,029.29 (33%)	122,239.87 (32.33%)
	Degree or above	35,576 (68.95%)	262,081 (69.32%)	18,484.42 (35.82%)	136,845.83 (36.2%)
Ethnic group	White	49,169 (95.29%)	362,519 (95.89%)	46,466.04 (90.05%)	345,488.53 (91.38%)
	Non-White	2431 (4.71%)	15,544 (4.11%)	5133.96 (9.95%)	32,574.47 (8.62%)
Household income	<£16k	6711 (13.01%)	49,188 (13.01%)	9209.31 (17.85%)	66,580.40 (17.61%)
	£16k - £30k	11,198 (21.7%)	84,173 (22.26%)	12,682.74 (24.58%)	96,549.04 (25.54%)
	£30k - £60k	16,459 (31.9%)	120,438 (31.86%)	15,043.87 (29.15%)	109,735.03 (29.03%)
	£60k -£90k	7353 (14.25%)	52,131 (13.79%)	5649.25 (10.95%)	39,855.49 (10.54%)
	£90k+	5131 (9.94%)	36,374 (9.62%)	3657.11 (7.09%)	25,646.75 (6.78%)
	Missing	4748 (9.2%)	35,759 (9.46%)	5357.72 (10.38%)	39,696.29 (10.5%)

### 2.2 Confidence in institutions

**Fig. 2:**
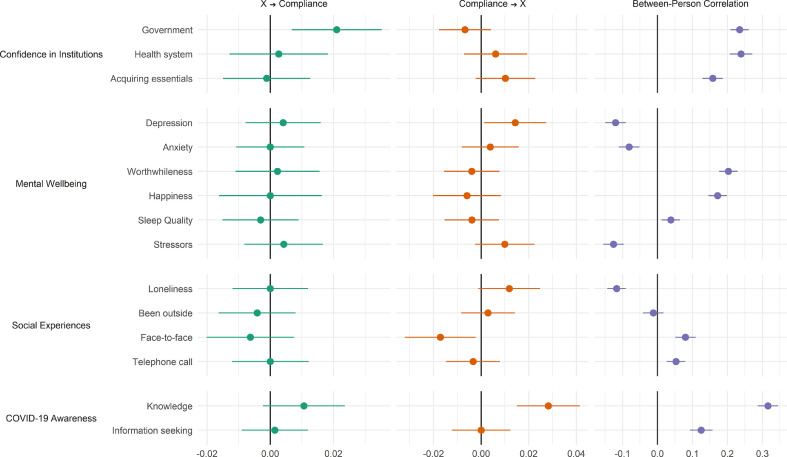
RI-CLPM Model Results. Left panel shows the cross-lagged effect of the exposure variable on self-reported compliance; the middle panel shows the cross-lagged effect of compliance on the exposure variable; and the right panel shows the correlation between the random intercept terms for the exposure and compliance (i.e. the between person correlations). Estimates are standardised.

When exploring cross-sectional “between” variation in self-reported compliance and confidence in institutions, higher confidence in government, the health service and access to essentials were all associated with higher compliance, although associations were small (right panel, Fig. 2). However, when exploring the cross-lagged associations, an increase in confidence in government was related to an increase in compliance at the next wave, but no other factors were statistically significantly related to compliance in later waves (left panel). Compliance did not predict changes in confidence in institutions (middle panel).

### 2.3 Mental wellbeing

Higher compliance was cross-sectionally related to lower depression, anxiety, and stressors, and higher happiness, sense that life's activities were worthwhile, and sleep quality (Fig. 2). However, when exploring the cross-lagged associations, no factors relating to mental wellbeing were associated with later improvements in compliance, and instead compliance was related to increases in depression.

### 2.4 Social experiences

Higher compliance was cross-sectionally related to lower loneliness and higher face-to-face and telephone contact but not to time spent outdoors (Fig. 2). However, when exploring the cross-lagged associations, no factors relating to social experiences were associated with later improvements in compliance, and instead compliance was related to decreases in face-to-face contact.

### 2.5 COVID-19 awareness

Higher compliance was cross-sectionally related to higher levels of knowledge and information seeking relating to COVID-19 (Fig. 2). However, when exploring the cross-lagged associations, no factors relating to COVID-19 awareness were associated with later improvements in compliance, and instead compliance was related to increases in knowledge about COVID-19.

The average autoregressive effect of compliance upon later compliance in the RI-CLPM models was 0•34, indicating that one third of the change in compliance persisted to the next wave and suggesting cross-lagged effects did not dissipate immediately.

### 2.6 Time use

**Fig. 3:**
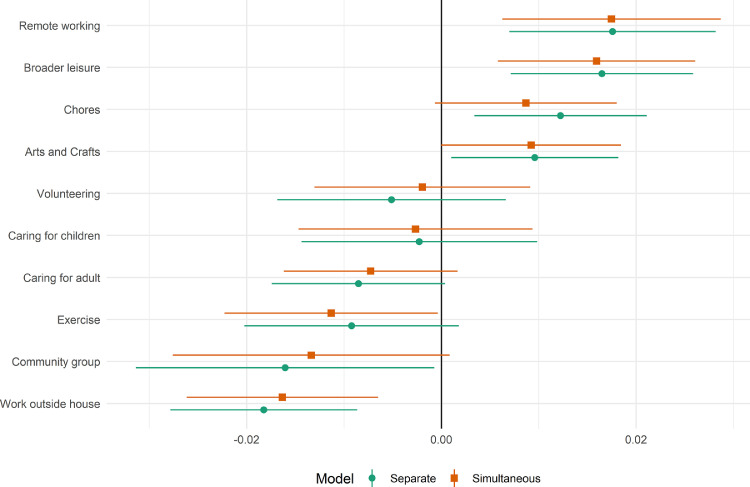
Standardised fixed effects model results, regression of compliance on time-use variables, entered separately or simultaneously into models.

We assessed concurrent changes between time use and compliance levels using fixed effect models. Estimates from these models are displayed in Fig. 3. Working outside the house, caring for a friend or relative, and engaging with a community group were each associated with lower compliance, while time spent in arts and crafts, broader leisure, doing household chores, and working remotely were associated with higher compliance. Associations were generally little impacted by whether time-use variables were added separately or simultaneously to models.

### 2.7 Sensitivity analyses

RI-CLPM estimates were generally similar when stratifying by gender (Supplementary Fig. S5). Exceptions were that depressive symptoms, lower happiness, and knowledge of COVID-19 were longitudinally related to higher compliance in females, with no clear association among males (though confidence intervals were wide). There was also an association between compliance and greater loneliness, depression and lower happiness at next wave among females. Supplementary Table S9 provides differences in the effect sizes (+ 95% CI) by gender. The results of fixed effects models using different adjustments for time trends showed that not accounting for time generated large biases in results, but estimates were very similar regardless of whether time was accounted for with linear trends, cubic trends or date fixed effects (Supplementary Fig. S6), suggesting our results are not confounded by time trends.

## 3 Discussion

This study explored predictors of self-reported compliance during the COVID-19 pandemic in a longitudinal sample of adults in the UK during the first three months of social distancing measures. Whilst there were a number of cross-sectional associations between studied factors and compliance, when looking at within-person changes, factors relating to mental health, confidence in the health service or access to essentials, social experiences, and awareness of COVID-19 were not related to future changes in compliance. The only factor that was associated with future compliance was confidence in government. Though associations were small, when looking at population-level behaviours, the impact of such associations can be important. This suggests that cross-sectional designs – the predominant approach used in the literature – could provide substantially biased results and highlights the importance of longitudinal data in investigating predictors of compliance during pandemics. When looking at how compliance affected other factors such as mental health, there was weak evidence that compliance was related to small later increases in depressive symptoms and loneliness, predominantly among women. But we did find evidence for parallel changes in compliance and behaviours, with individuals reporting low compliance also reporting spending more time working outside the house, participating in community groups or caring for an adult, and individuals reporting high compliance also reporting more remote working.

Our finding that trust in government predicted self-reported compliance echoes findings from previous studies [3,5, 6, 7]. While the estimated effects are small, changes in compliance did not dissipate immediately, with one third of the change persisting to the next data collection. Further, at a population level, the consequences of small changes in compliance could be considerable, particularly if there is “social contagion” in compliance levels. Governments should be aware that decisions that undermine public confidence may also undermine efforts to tackle the pandemic. Previous work has shown that confidence in the central UK government fell after the announcement to relax the first lockdown and following the decision to keep a senior advisor in post after reports he had broken lockdown rules [30]. Our results suggest these decisions may have had adverse consequences for the managing the pandemic.

It is notable that we found little clear evidence of an association between mental health and wellbeing and compliance. This is at odds with some previous studies, which have suggested associations, albeit often in opposite directions [10,13,17,18]. One possibility is that anxiety, depression and wellbeing have multiple, countervailing effects on compliance behaviours, meaning that the net association is context specific. For instance, a recent study found little bivariate association between depressive symptoms and compliance, but a negative correlation once COVID-19 related fears were added into regression models [13]. Another possibility is that previous studies (many of which have use cross-sectional data) capture reverse effects, with compliance predicting worsening of mental health. We found some evidence that compliance could lead to worse mental health, particularly among women. In considering the pathway through which this finding may have occurred, compliance was also related to increased loneliness in women, which is associated with higher depressive symptoms. Nevertheless the associations observed here are small, so such effects, if they are occurring, may not be of clinical relevance. However, it is possible that our estimates mask a wide degree of variation. A large literature shows heterogeneous responses to many major life stressors [31] and the costs of compliance are unlikely to be uniform. Therefore, future research should explore heterogeneity in both the determinants and consequences of complying with lockdown measures.

We also found little evidence that social isolation, measured as loneliness or days without contact, has an association with compliance. This is promising given that compliance entails lower close social contact. Again, though, it may be that average effects mask considerable heterogeneity and that net associations combine multiple countervailing effects. It is also possible that more complex modelling of cross-lagged effects – for instance using higher-order lags or accumulative measures of loneliness (and other factors) – may generate different results. Finally, this study showed that compliance is related to time use. The evidence that caring for friends or relatives and working outside the house is related to lower compliance (and conversely that remote working is related to higher compliance) may suggest that efforts are required to support those with care needs, improve or enforce guidelines in workplaces and financially support those whose workplaces are unsafe (e.g. through furlough schemes), though non-compliance may also result from entering other environments when providing care or working, such as commuting on public transport. It is also notable that many leisure activities were related to higher levels of compliance, though the direction of this association is unclear. Engagement in leisure may help to reduce boredom, which is a common experience during quarantines and an important barrier to compliance [19,32], but a consequence of higher compliance is also likely to be that individuals have more time for leisure. It is notable that among women, compliance was related to feeling one's activities were less worthwhile and also to increased feelings of loneliness. This suggests there could be value to supporting safe socially-distanced opportunities for leisure engagement to help individuals find worthwhile, social activities to undertake during the pandemic. This may be a particular benefit to individuals who have lost employment as they may have access to fewer purposeful activities.

This study had a number of strengths. The longitudinal design allowed us to account for two possible sources of bias: confounding via time-invariant between-person characteristics and confounding through reverse causality. While many variables shared common secular time trends, it appears that we were able to account for these sufficiently in our statistical modelling. By analysing multiple variables that displayed common secular trends, we were also provided with a second test of whether common time trends explained results. Together, our results are more supportive of a causal interpretation, though as we use observational data, it is still possible that results are explained by unobserved time-varying factors, such as changes in caseloads or fears of COVID-19. Another strength of this study was the length of follow-up. Previous studies have used much shorter time frames and typically focused on the earlier stages of the pandemic, when enforcement was stricter and compliance was higher, on average, across the population.

However, this study also had several limitations. First, we relied upon self-report measures, notably a single generic item of self-reported compliance with COVID-19 guidelines that has not been validated. While the salience of the pandemic may enhance recall, the opportunities for non-compliance are many and people may not remember specific instances of non-compliance (e.g., forgetting to sanitize hands in shops). Further, if guidelines are seen as too weak, it is possible that low confidence in government could have led participants to report low compliance even without changing behaviour. Though, if they were exceeding guidelines, they should still report full compliance. Future studies are encouraged that look at specific behaviours. Even though the study was anonymous and online, meaning participants could log self-reported compliance without fear of interviewer judgement, responses may be influenced by social desirability concerns. Less compliant individuals are also likely to be less knowledgeable about COVID-19 guidelines, particularly as they have been updated regularly, and so may be unable to accurately judge their own non-compliance (although this would not preclude the measure capturing participants’ beliefs about their own compliance). These factors likely bias towards finding smaller associations. The measure of compliance may also contain non-differential measurement error, which would again bias associations towards the null.

Another limitation of our study is the possibility of selection bias. We used data from a study set-up explicitly to research COVID-19. Though we used weighting, the sample was not representative of the general population. It is likely that individuals who participated – and remained in – the study had a higher interest in helping tackle the pandemic. This interest may manifest as a higher propensity to comply with guidelines. Another issue is that government guidelines became less stringent across the study period. Participants may have been more compliant than reported if they were unaware of current guidance. It is notable that time spent seeking COVID-19 related information declined markedly through time (Supplementary Fig. S2).

Nonetheless, this study still provides the largest longitudinal exploration of predictors of compliance during the COVID-19 pandemic to date, with important implications for policy makers. In particular, the results highlight the central role of trust in determining adherence to guidelines, showing that the actions of policy makers are not just of political relevance during pandemics but are also of public health relevance as they could have had wider impacts on compliance. Confidence in the central UK government to handle the pandemic effectively has fallen markedly across the pandemic, but in other countries – including Scotland – opinions of government effectiveness have increased or remained at high levels [30] (see Supplementary Fig. S7). This highlights that increasing citizen's trust is within governmental control. It is vital that governments work to engage with the public and communicate plans and rules effectively to improve trust and, consequently, that social distancing rules are followed as countries enter second waves.

## Author Contributions

LW and DS planned the data analysis. LW carried out the data analysis. LW, DS, and AS wrote the manuscript.

## Data sharing statement

Data from the COVID-19 Social Study will be made available at the end of the 2021. Code to replicate the analysis in this paper is available at https://osf.io/7y9pw/.

## Funding

This Covid-19 Social Study was funded by the 10.13039/501100000279Nuffield Foundation [WEL/FR-000022583], but the views expressed are those of the authors and not necessarily the Foundation. The study was also supported by the MARCH Mental Health Network funded by the Cross-Disciplinary Mental Health Network Plus initiative supported by UK Research and Innovation [ES/S002588/1], and by the 10.13039/100004440Wellcome Trust [221400/Z/20/Z]. DF was funded by the 10.13039/100010269Wellcome Trust [205407/Z/16/Z]. The researchers are grateful for the support of a number of organisations with their recruitment efforts including: the UKRI Mental Health Networks, Find Out Now, UCL BioResource, SEO Works, FieldworkHub, and Optimal Workshop. The study was also supported by HealthWise Wales, the Health and Car Research Wales initiative, which is led by Cardiff University in collaboration with SAIL, Swansea University.

## Declaration of Interests

The authors have no interests to declare.

## Appendix Supplementary materials

Image, application 1
