# Supplementary material for: Predictors of self-reported adherence to COVID-19 guidelines. A longitudinal observational study of 51,600 UK adults
Source: Lancet Reg Health Eur. 2021 Feb 26;4:100061. doi: 10.1016/j.lanepe.2021.100061 (PMC7907734; doi:10.1016/j.lanepe.2021.100061)
Supplement: Supplementary file 1 [file mmc1.docx]

# SUPPLEMENTARY INFORMATION

## MEASURES

#### Mental Health and Wellbeing

Depression during the past week was measured using the Patient Health Questionnaire (PHQ-9); a standard 9-item instrument for diagnosing depression in primary care, with 4-point responses ranging from “not at all” to “nearly every day” (range 0-27; higher scores indicate more depressive symptoms).

Anxiety during the past week was measured using the Generalised Anxiety Disorder assessment (GAD-7); a well-validated 7-item tool used to screen and diagnose generalised anxiety disorder in clinical practice and research, with 4-point responses ranging from “not at all” to “nearly every day” (range 0-21; higher scores indicate more symptoms of anxiety).

Meaning in life and happiness were measured with a two single item measures: “In the past week, to what extent have you felt the things you are doing in your life are worthwhile?”; “In the past week, how happy did you feel?”. Both were measured on an 11-point scale (0 = not at all; 10 = completely) and are drawn from the UK Office for National Statistics’ Annual Population Survey which is used by the UK Government to measure national subjective wellbeing ^1^.

Sleep quality was measured with a single item measure, “Over the past week, how has your sleep been?”. The item was measured on a five-point Likert scale: 1 = Very good, 2 = Good, 3 = Average, 4 = Not good, 5 = Very poor. We reverse code this so higher scores into better quality sleep.

Stressors were captured from two questions that asked participants to select which of a list of items had caused them (a) stress (however minor) in the past week, or (b) significant stress in the past week. We create an index of worries as the sum of endorsements for: “your own safety/security”, “finances”, “losing your job/unemployment”, “getting food”, and “getting medication”, “future plans”, “boredom”. The index ranges 0-7.

#### Social Experiences

Isolation was measured as number of days over the past week that the participant had: not left the house or garden (Home Isolation), had had face-to-face contact for 15 minutes or more (Face-to-face Isolation), and had had a phone or video call contact with someone for 15 minutes or more (Phone Isolation).

Loneliness was measured with the 3-item UCL-3 Loneliness scale, a short version of the Revise UCL Loneliness Scale. The items are: “How often do you feel that you lack companionship?”, “How often do you feel left out?”, and “How often do you feel isolated from others?”. Each was measured on a three-point scale – 1 = hardly ever, 2 = some of the time, 3 = often. We use the sum score (range 3-9). Higher scores indicate greater loneliness.

#### Confidence in Institutions

Confidence in (devolved) government was measured between 21 March – 18 June using a single-item measure: “How much confidence do you have in the UK government that they can handle Covid-19 well?”. Responses to both questions were scored on a 1 (“None at all”) to 7 (“Lots”) scale. Participants from Scotland, Wales and Northern Ireland were prompted to respond about their devolved government, specifically. From 18 June, participants were instead asked separate questions on confidence in central government and confidence in their devolved government, which were scored on the same seven-point scale as previously. We used responses on central government for participants from England and responses on devolved government, otherwise.

Confidence in the health system and confidence in acquiring essentials was measured with two questions: “How much confidence do you have that the UK health service can cope during Covid-19?”; and “How much confidence do you have that essentials (e.g. access to food, water, medicines, deliveries) will be maintained during Covid-19?”. Participants from Scotland, Wales and Northern Ireland were asked to answer about the health service in their home nation, specifically. Responses to both questions were scored on a 1 (“None at all”) to 7 (“Lots”) scale.

#### COVID-19 Awareness

Knowledge was measured with a single item question, “How would you rate your knowledge level on Covid-19?”. Response were scored on a seven-point scale (1 = very poor knowledge, 7 = very good knowledge). Information seeking was measured with two items on time spent reading, watching the news, or listening to radio broadcasts about COVID-19 and time spend tweeting, blogging, or posting content online about COVID-19. Both were measured on a five-point scale (0 = did not do; 1 = <30 minutes; 2 = 30 minutes – 2 hours; 3 = 3-5 hours; 4 = 6+ hours). We used the average of responses to the two questions.

### Time Use

Each week, participants were asked how much time they had spent in each of a list of activities during the weekday prior to interview. Time spent on each activity was collected using a five-point scale (0 = did not do; 1 = <30 minutes; 2 = 30 minutes – 2 hours; 3 = 3-5 hours; 4 = 6+ hours). To measure time spent in work, we included separate measures for time spent remote working, working outside house, caring for friends or relatives, childcare, volunteer work and household chores. To measure time spent in leisure activities, we included separate measures for community group engagement, arts and crafts, gardening, and broader leisure (including TV, video, and internet use). The specific questionnaire items used to construct these measures are displayed below. Where we used multiple items to measure a single activity, we used the average score across items.

| **Activity** | **Questionnaire Items** |
| --- | --- |
| *Remote working* | - Phoning or video talking with colleagues whilst working remotely  - Undertaking other work remotely |
| *Working outside house* | - Going to work outside of the house (e.g. to the office) |
| *Caring for friends or relatives* | - Caring for a friend or relative |
| *Childcare* | - Caring for children (e.g. bathing, feeding, doing homework with etc)  - Playing with children (e.g. general play or board games or card games) |
| *Volunteer work* | - Volunteering |
| *Household chores* | - Household chores (cooking, cleaning, ironing, tidying, online shopping etc) |
| *Exercise* | - Going out for a walk or other gentle physical activity  - Going out for moderate or high intensity activity (e.g. running, cycling or swimming) |
| *Community group engagement* | - Going out of the house to engage in a community group |
| *Arts and crafts* | - Engaging in a home-based arts or crafts activity (e.g. painting, creative writing, sewing, playing music, etc)  - Engaging in a digital arts activity (e.g. streaming a concert, virtual tour of a museum etc)  - Doing DIY, woodwork, metal work, model making or similar |
| *Broader leisure* | - Playing video or computer games alone, or with adults or children  - Watching TV, films, Netflix etc (NOT for information on Covid-19)  - Browsing the internet (NOT for information on Covid-19)  - Procrastinating or not doing anything in particular |

Time trends for compliance and the time use and non-time use measures are displayed in Figures S2 and S3. There is strong time trends in many of the measures, notably compliance and confidence in government, both of which fell over the analysis period. Many of the measures also display rhythmic patterns. We explore this in further detail by estimating random effects within-between models (Figures S8 and S9) ^2^. These show the variation in each measure by day of response, due to particular individuals being more likely to answer of certain days of the week (between-person variation) and people being more likely to give particular responses on certain days of the week (within-person variation). Several of the measures differ by day of response. Notably, self-reported compliance is lower at weekends. We should also note that fewer people choose to respond to the survey at weekends (data not shown).

## STATISTICAL ANALYSIS

RI-CLPM models were fit using the lavaan R package version 0.6-6 ^3^. To correct confidence intervals for multiple testing, we used Bonferroni-corrected confidence intervals (15 comparisons for non-time-use measures [α = 0.05/15 = 0.0033] and 10 comparisons for time-use measures [α = 0.05/10 = 0.005]). As the COVID-19 Social Study used a nonprobability sampling design, we weighted data using cross-sectional weights. The weights were created using the Stata user written command ‘ebalance’. The weighted data were matched to population statistics across the following characteristics: age, gender, ethnicity, education, and country of living. Population statistics were taken from the ONS’s Annual Population Survey ^4^.

We used an unbalanced panel in the fixed effects and RI-CLPM models. An issue with using an unbalanced panel is that participants who remained in the survey longer contribute more data. Non-random attrition from the study may create biases, though this is partly offset by our use of within-variation when estimating cross-lagged effects. In Figures S10, we assessed attrition from the survey in detail. We ran linear OLS regression models to investigate baseline characteristics of participants who dropped out the survey by the end of the analysis period. We also ran fixed effects models to investigate within-person changes in participant characteristics (e.g. mental health) in the last interview completed before attrition from the survey. Individuals who dropped out from the study complied with government guidelines less, experienced more stressors, had lower confidence in government and had worse mental health and lower subjective wellbeing. However, in their last interviews’, participants reported more confidence in government and better mental health and subjective wellbeing than their (participant-specific) average level. Lower compliance with guidelines was related to dropping out in both OLS and fixed effects regressions. Figure S4 shows average compliance levels by number of waves completed. Table S3 shows demographic characteristics by number of waves completed. Young people are more likely to complete fewer waves.

Our main RI-CLPM models included linear time trends to account for secular trends in several of the studied factors (Figures S2 and S3). We attempted more complex adjustments for time, including cubic times trends and date fixed effects, but models did not converge. Consequently, as an alternative sensitivity analysis, we instead ran fixed effects models using different adjustments for time trends. Not accounting for time was found to generate large biases in results, but estimates were very similar regardless of whether time was accounted for with linear trends, cubic trends or date fixed effects (Figure S6). Note, the RI-CLPM model results are preferable to the fixed effects results as RI-CLPM models allow for modelling the endogeneity of the explanatory variables. A comparison of the RI-CLPM and fixed effects results is shown in Figure S11). The time period differs slightly between follow-ups, but is generally between 7-9 days.

Data from the COVID-19 Social Study will be made available at the end of the 2021. The code used in this analysis is available at <https://osf.io/7y9pw/>.

## RESULTS

Fit statistics for the RI-CLPM models are displayed in Table S6. While RMSEA statistics are lower than conventional cut-offs, SRMR, CFI and TLI scores are poor in most cases. This is likely to be partly due to the non-normally distributed variables used in this analysis. The average autoregressive effect of compliance upon later compliance in the RI-CLPM models was 0.34, indicating that one third of the change in compliance persisted to the next interview and suggesting cross-lagged effects did not dissipate immediately. Model coefficients for the RI-CLPM and fixed effects models are displayed in Tables S7 and S8. The difference in the RI-CLPM coefficients between males and females are displayed in Table S9.

## TABLES

**Table S2**: (Unweighted) descriptive statistics, time-varying variables

|  | Variable | Mean | Total SD^1^ | Within SD^2^ | Between SD^3^ | Residual Within SD^4^ |
| --- | --- | --- | --- | --- | --- | --- |
|  | Adherence to guidelines | 6.456 | 0.85 | 0.54 | 0.67 | 0.52 |
| Confidence in Institutions | Government | 3.991 | 1.84 | 0.87 | 1.62 | 0.79 |
|  | Health system | 5.322 | 1.35 | 0.71 | 1.18 | 0.69 |
|  | Acquiring essentials | 5.737 | 1.17 | 0.64 | 1.01 | 0.62 |
| Mental Wellbeing | Depression | 5.896 | 5.68 | 2.19 | 5.44 | 2.18 |
|  | Anxiety | 4.419 | 4.99 | 1.98 | 4.78 | 1.97 |
|  | Worthwhileness | 6.169 | 2.48 | 1.22 | 2.20 | 1.22 |
|  | Happiness | 6.021 | 2.31 | 1.00 | 2.12 | 1.00 |
|  | Sleep Quality | 3.121 | 1.04 | 0.55 | 0.91 | 0.55 |
|  | Stressors | 1.412 | 1.39 | 0.76 | 1.22 | 0.75 |
| Social Experiences | Loneliness | 4.814 | 1.92 | 0.73 | 1.80 | 0.73 |
|  | Been outside | 5.640 | 1.85 | 1.12 | 1.60 | 1.10 |
|  | Face-to-face | 6.209 | 1.77 | 0.85 | 1.82 | 0.85 |
|  | Telephone call | 4.286 | 2.09 | 1.11 | 1.84 | 1.10 |
| COVID-19 Awareness | Knowledge | 5.520 | 1.07 | 0.48 | 0.97 | 0.48 |
|  | Information seeking | 0.757 | 0.62 | 0.39 | 0.51 | 0.36 |
| Time Use | Remote working | 0.976 | 1.21 | 0.56 | 1.10 | 0.56 |
|  | Work outside house | 0.427 | 1.16 | 0.65 | 1.01 | 0.65 |
|  | Caring for adult | 0.351 | 0.95 | 0.49 | 0.84 | 0.49 |
|  | Volunteering | 0.175 | 0.61 | 0.37 | 0.50 | 0.37 |
|  | Chores | 1.857 | 0.86 | 0.54 | 0.70 | 0.54 |
|  | Caring for children | 0.411 | 0.94 | 0.28 | 0.99 | 0.28 |
|  | Exercise | 0.744 | 0.70 | 0.41 | 0.59 | 0.41 |
|  | Community group | 0.014 | 0.17 | 0.14 | 0.11 | 0.14 |
|  | Arts and Crafts | 0.299 | 0.46 | 0.29 | 0.39 | 0.29 |
|  | Broader leisure | 1.330 | 0.62 | 0.35 | 0.54 | 0.34 |
| ^1^ “Total SD” is the total standard deviation for a given row variable across all observations.  ^2^ “Within SD” is the standard deviation from participant-specific mean values.  ^3^ “Between SD” is the standard deviation of participant specific mean values.  ^4 “^Residual within SD” is the average within-person standard deviation for the residual of a given row variable, extracted from mixed effect regression models which include a linear time trend and a random intercept for each participant – i.e., it captures the variation in a variable once time trends and person-level differences are accounted for. | | | | | | |

**Table S3**: (Unweighted) descriptive statistics by number of waves of data collection.

|  | Variable | 1/Missing Weight | 2 | 3 | 4 | 5 | 6 | 7 | 8 | 9 | 10 | 11+ |
| --- | --- | --- | --- | --- | --- | --- | --- | --- | --- | --- | --- | --- |
|  | n | 21,740 | 6,239 | 4,518 | 3,621 | 2,906 | 3,089 | 3,756 | 2,707 | 4,123 | 7,623 | 13,018 |
| Gender | Male | 5,653 (26%) | 1,513 (24.25%) | 1,016 (22.49%) | 886 (24.47%) | 700 (24.09%) | 707 (22.89%) | 829 (22.07%) | 618 (22.83%) | 1,078 (26.15%) | 2,037 (26.72%) | 3,353 (25.76%) |
|  | Female | 15,732 (72.36%) | 4,726 (75.75%) | 3,502 (77.51%) | 2,735 (75.53%) | 2,206 (75.91%) | 2,382 (77.11%) | 2,927 (77.93%) | 2,089 (77.17%) | 3,045 (73.85%) | 5,586 (73.28%) | 9,665 (74.24%) |
|  | <NA> | 355 (1.63%) |  |  |  |  |  |  |  |  |  |  |
| Country of residence | England | 18,221 (83.81%) | 5,087 (81.54%) | 3,589 (79.44%) | 2,980 (82.3%) | 2,438 (83.9%) | 2,639 (85.43%) | 3,277 (87.25%) | 2,038 (75.29%) | 2,604 (63.16%) | 5,886 (77.21%) | 11,066 (85.01%) |
|  | Wales | 1,700 (7.82%) | 660 (10.58%) | 543 (12.02%) | 350 (9.67%) | 249 (8.57%) | 269 (8.71%) | 292 (7.77%) | 496 (18.32%) | 1,264 (30.66%) | 1,152 (15.11%) | 889 (6.83%) |
|  | Scotland | 1,493 (6.87%) | 412 (6.6%) | 325 (7.19%) | 251 (6.93%) | 186 (6.4%) | 153 (4.95%) | 160 (4.26%) | 143 (5.28%) | 218 (5.29%) | 519 (6.81%) | 929 (7.14%) |
|  | Northern Ireland | 326 (1.5%) | 80 (1.28%) | 61 (1.35%) | 40 (1.1%) | 33 (1.14%) | 28 (0.91%) | 27 (0.72%) | 30 (1.11%) | 37 (0.9%) | 66 (0.87%) | 134 (1.03%) |
| Age group | 18-29 | 3,759 (17.29%) | 919 (14.73%) | 523 (11.58%) | 394 (10.88%) | 283 (9.74%) | 244 (7.9%) | 264 (7.03%) | 207 (7.65%) | 244 (5.92%) | 375 (4.92%) | 628 (4.82%) |
|  | 30-45 | 8,317 (38.26%) | 2,428 (38.92%) | 1,676 (37.1%) | 1,219 (33.66%) | 909 (31.28%) | 884 (28.62%) | 954 (25.4%) | 740 (27.34%) | 981 (23.79%) | 1,837 (24.1%) | 2,762 (21.22%) |
|  | 46-59 | 5,705 (26.24%) | 1,879 (30.12%) | 1,445 (31.98%) | 1,173 (32.39%) | 970 (33.38%) | 1,100 (35.61%) | 1,252 (33.33%) | 886 (32.73%) | 1,301 (31.55%) | 2,539 (33.31%) | 4,051 (31.12%) |
|  | 60+ | 3,959 (18.21%) | 1,013 (16.24%) | 874 (19.34%) | 835 (23.06%) | 744 (25.6%) | 861 (27.87%) | 1,286 (34.24%) | 874 (32.29%) | 1,597 (38.73%) | 2,872 (37.68%) | 5,577 (42.84%) |
| Highest qualification | GCSE or below | 4,112 (18.91%) | 894 (14.33%) | 629 (13.92%) | 536 (14.8%) | 360 (12.39%) | 392 (12.69%) | 482 (12.83%) | 329 (12.15%) | 557 (13.51%) | 1,048 (13.75%) | 1,778 (13.66%) |
|  | A-levels or equivalent | 4,397 (20.23%) | 1,236 (19.81%) | 824 (18.24%) | 659 (18.2%) | 471 (16.21%) | 478 (15.47%) | 635 (16.91%) | 493 (18.21%) | 676 (16.4%) | 1,339 (17.57%) | 2,208 (16.96%) |
|  | Degree or above | 13,231 (60.86%) | 4,109 (65.86%) | 3,065 (67.84%) | 2,426 (67%) | 2,075 (71.4%) | 2,219 (71.84%) | 2,639 (70.26%) | 1,885 (69.63%) | 2,890 (70.09%) | 5,236 (68.69%) | 9,032 (69.38%) |
| Ethnic group | White | 19,527 (89.82%) | 5,792 (92.84%) | 4,260 (94.29%) | 3,409 (94.15%) | 2,726 (93.81%) | 2,913 (94.3%) | 3,591 (95.61%) | 2,570 (94.94%) | 3,960 (96.05%) | 7,354 (96.47%) | 12,594 (96.74%) |
|  | Non-White | 1,935 (8.9%) | 447 (7.16%) | 258 (5.71%) | 212 (5.85%) | 180 (6.19%) | 176 (5.7%) | 165 (4.39%) | 137 (5.06%) | 163 (3.95%) | 269 (3.53%) | 424 (3.26%) |
|  | <NA> | 278 (1.28%) |  |  |  |  |  |  |  |  |  |  |
| Household income | <£16k | 3,609 (16.6%) | 835 (13.38%) | 595 (13.17%) | 468 (12.92%) | 375 (12.9%) | 384 (12.43%) | 445 (11.85%) | 361 (13.34%) | 520 (12.61%) | 1,009 (13.24%) | 1,719 (13.2%) |
|  | £16k - £30k | 4,593 (21.13%) | 1,255 (20.12%) | 928 (20.54%) | 751 (20.74%) | 542 (18.65%) | 625 (20.23%) | 802 (21.35%) | 572 (21.13%) | 999 (24.23%) | 1,730 (22.69%) | 2,994 (23%) |
|  | £30k - £60k | 6,413 (29.5%) | 1,973 (31.62%) | 1,450 (32.09%) | 1,165 (32.17%) | 926 (31.87%) | 993 (32.15%) | 1,221 (32.51%) | 874 (32.29%) | 1,331 (32.28%) | 2,410 (31.61%) | 4,116 (31.62%) |
|  | £60k -£90k | 2,943 (13.54%) | 976 (15.64%) | 700 (15.49%) | 526 (14.53%) | 463 (15.93%) | 487 (15.77%) | 554 (14.75%) | 388 (14.33%) | 533 (12.93%) | 1,027 (13.47%) | 1,699 (13.05%) |
|  | £90k+ | 2,099 (9.66%) | 687 (11.01%) | 473 (10.47%) | 376 (10.38%) | 343 (11.8%) | 336 (10.88%) | 380 (10.12%) | 258 (9.53%) | 368 (8.93%) | 700 (9.18%) | 1,210 (9.29%) |
|  | Missing | 2,083 (9.58%) | 513 (8.22%) | 372 (8.23%) | 335 (9.25%) | 257 (8.84%) | 264 (8.55%) | 354 (9.42%) | 254 (9.38%) | 372 (9.02%) | 747 (9.8%) | 1,280 (9.83%) |

**Table S4**: (Unweighted) descriptive statistics. Sex and age.

|  | Male | | Female | |
| --- | --- | --- | --- | --- |
| Age Group | N | Observations | N | Observations |
| 18-29 | 657 (5.16%) | 3,759 (3.95%) | 3,424 (8.81%) | 20,461 (7.23%) |
| 30-45 | 2,706 (21.25%) | 17,303 (18.2%) | 11,684 (30.06%) | 77,485 (27.38%) |
| 46-59 | 3,702 (29.06%) | 26,604 (27.99%) | 12,894 (33.18%) | 95,143 (33.62%) |
| 60+ | 5,672 (44.53%) | 47,396 (49.86%) | 10,861 (27.95%) | 89,912 (31.77%) |

**Table S5**: (Unweighted) descriptive statistics. Detailed ethnic group.

| Ethnic Group | Individuals (%) | Observations (%) |
| --- | --- | --- |
| White | 49,169 (95.29%) | 362,519 (95.89%) |
| Asian/Asian British | 714 (1.38%) | 4,474 (1.18%) |
| Black/Black British | 342 (0.66%) | 2,040 (0.54%) |
| Mixed Race | 822 (1.59%) | 5,375 (1.42%) |
| Chinese/Chinese British | 170 (0.33%) | 1,138 (0.3%) |
| Middle Eastern/Middle Eastern British | 111 (0.22%) | 609 (0.16%) |
| Other Ethnic Group | 272 (0.53%) | 1,908 (0.5%) |

**Table S6:** Fit Statistics, RI-CLPM models.

|  | Variable | CFI | TLI | RMSEA | SRMR | N |
| --- | --- | --- | --- | --- | --- | --- |
| Confidence in Institutions | Government | 0.936 | 0.937 | 0.043 | 0.122 | 51600 |
|  | Health system | 0.936 | 0.937 | 0.039 | 0.083 | 51600 |
|  | Acquiring essentials | 0.933 | 0.934 | 0.039 | 0.08 | 51600 |
| Mental Wellbeing | Depression | 0.971 | 0.971 | 0.035 | 0.08 | 51594 |
|  | Anxiety | 0.97 | 0.97 | 0.034 | 0.077 | 51593 |
|  | Worthwhileness | 0.949 | 0.95 | 0.035 | 0.078 | 51600 |
|  | Happiness | 0.967 | 0.967 | 0.028 | 0.073 | 47380 |
|  | Sleep Quality | 0.952 | 0.953 | 0.033 | 0.076 | 51600 |
|  | Stressors | 0.943 | 0.944 | 0.035 | 0.08 | 51600 |
| Social Experiences | Loneliness | 0.957 | 0.957 | 0.037 | 0.078 | 51591 |
|  | Been outside | 0.926 | 0.927 | 0.037 | 0.093 | 51291 |
|  | Face-to-face | 0.949 | 0.949 | 0.031 | 0.075 | 49926 |
|  | Telephone call | 0.937 | 0.937 | 0.035 | 0.08 | 50673 |
| COVID-19 Awareness | Knowledge | 0.941 | 0.941 | 0.04 | 0.084 | 51600 |
|  | Information seeking | 0.933 | 0.934 | 0.035 | 0.081 | 51582 |

**Table S7:** Model Results, RI-CLPM models.

| Sex |  | Variable | N | X ➔ Compliance | Compliance ➔ X | Between-Person Correlation |
| --- | --- | --- | --- | --- | --- | --- |
| All | Confidence in Institutions | Government | 51,600 | 0.021 (0.007, 0.035) | -0.007 (-0.018, 0.004) | 0.235 (0.209, 0.261) |
|  |  | Health system | 51,600 | 0.003 (-0.013, 0.018) | 0.006 (-0.007, 0.019) | 0.239 (0.207, 0.271) |
|  |  | Acquiring essentials | 51,600 | -0.001 (-0.015, 0.013) | 0.01 (-0.002, 0.023) | 0.158 (0.129, 0.187) |
|  | Mental Wellbeing | Depression | 51,594 | 0.004 (-0.008, 0.016) | 0.014 (0.001, 0.027) | -0.12 (-0.149, -0.091) |
|  |  | Anxiety | 51,593 | 0 (-0.011, 0.011) | 0.004 (-0.008, 0.016) | -0.081 (-0.11, -0.052) |
|  |  | Worthwhileness | 51,600 | 0.002 (-0.011, 0.016) | -0.004 (-0.016, 0.008) | 0.203 (0.177, 0.229) |
|  |  | Happiness | 47,380 | 0 (-0.016, 0.016) | -0.006 (-0.02, 0.008) | 0.172 (0.146, 0.198) |
|  |  | Sleep Quality | 51,600 | -0.003 (-0.015, 0.009) | -0.004 (-0.015, 0.008) | 0.038 (0.012, 0.064) |
|  |  | Stressors | 51,600 | 0.004 (-0.008, 0.017) | 0.01 (-0.003, 0.022) | -0.126 (-0.155, -0.097) |
|  | Social Experiences | Loneliness | 51,591 | 0 (-0.012, 0.012) | 0.012 (-0.001, 0.025) | -0.117 (-0.143, -0.091) |
|  |  | Been outside | 51,291 | -0.004 (-0.016, 0.008) | 0.003 (-0.008, 0.014) | -0.012 (-0.041, 0.017) |
|  |  | Face-to-face | 49,926 | -0.006 (-0.02, 0.008) | -0.017 (-0.032, -0.002) | 0.08 (0.051, 0.109) |
|  |  | Telephone call | 50,673 | 0 (-0.012, 0.012) | -0.003 (-0.015, 0.008) | 0.053 (0.027, 0.079) |
|  | COVID-19 Awareness | Knowledge | 51,600 | 0.011 (-0.002, 0.024) | 0.028 (0.015, 0.042) | 0.316 (0.287, 0.345) |
|  |  | Information seeking | 51,582 | 0.001 (-0.009, 0.012) | 0 (-0.012, 0.012) | 0.125 (0.093, 0.157) |
| Female | Confidence in Institutions | Government | 38,863 | 0.023 (0.008, 0.037) | -0.007 (-0.018, 0.003) | 0.224 (0.198, 0.25) |
|  |  | Health system | 38,863 | -0.001 (-0.013, 0.01) | 0.002 (-0.012, 0.015) | 0.224 (0.195, 0.253) |
|  |  | Acquiring essentials | 38,863 | -0.004 (-0.017, 0.01) | 0.009 (-0.004, 0.021) | 0.135 (0.103, 0.167) |
|  | Mental Wellbeing | Depression | 38,859 | 0.012 (0, 0.024) | 0.026 (0.011, 0.04) | -0.142 (-0.174, -0.11) |
|  |  | Anxiety | 38,858 | 0.004 (-0.007, 0.014) | 0.009 (-0.005, 0.022) | -0.093 (-0.122, -0.064) |
|  |  | Worthwhileness | 38,863 | -0.005 (-0.018, 0.009) | -0.02 (-0.034, -0.006) | 0.198 (0.169, 0.227) |
|  |  | Happiness | 35,746 | -0.013 (-0.024, -0.002) | -0.025 (-0.042, -0.007) | 0.164 (0.135, 0.193) |
|  |  | Sleep Quality | 38,863 | -0.004 (-0.016, 0.008) | -0.007 (-0.018, 0.005) | 0.021 (-0.008, 0.05) |
|  |  | Stressors | 38,863 | 0.006 (-0.007, 0.018) | 0.012 (0.002, 0.022) | -0.142 (-0.171, -0.113) |
|  | Social Experiences | Loneliness | 38,856 | 0.009 (-0.002, 0.021) | 0.024 (0.011, 0.037) | -0.119 (-0.145, -0.093) |
|  |  | Been outside | 38,632 | -0.008 (-0.02, 0.004) | -0.001 (-0.014, 0.011) | -0.01 (-0.042, 0.022) |
|  |  | Face-to-face | 37,779 | -0.008 (-0.022, 0.006) | -0.02 (-0.035, -0.005) | 0.045 (0.016, 0.074) |
|  |  | Telephone call | 38,348 | -0.002 (-0.014, 0.01) | -0.004 (-0.017, 0.008) | 0.046 (0.02, 0.072) |
|  | COVID-19 Awareness | Knowledge | 38,863 | 0.017 (0.004, 0.03) | 0.04 (0.026, 0.053) | 0.335 (0.306, 0.364) |
|  |  | Information seeking | 38,851 | -0.001 (-0.011, 0.01) | -0.004 (-0.016, 0.008) | 0.157 (0.128, 0.186) |
| Male | Confidence in Institutions | Government | 12,737 | 0.019 (-0.004, 0.043) | -0.007 (-0.025, 0.011) | 0.243 (0.202, 0.284) |
|  |  | Health system | 12,737 | 0.009 (-0.018, 0.036) | 0.01 (-0.015, 0.034) | 0.271 (0.218, 0.324) |
|  |  | Acquiring essentials | 12,737 | 0 (-0.024, 0.024) | 0.013 (-0.01, 0.035) | 0.186 (0.139, 0.233) |
|  | Mental Wellbeing | Depression | 12,735 | -0.008 (-0.044, 0.028) | 0.005 (-0.016, 0.026) | -0.142 (-0.192, -0.092) |
|  |  | Anxiety | 12,735 | -0.004 (-0.036, 0.029) | -0.001 (-0.019, 0.017) | -0.115 (-0.168, -0.062) |
|  |  | Worthwhileness | 12,737 | 0.014 (-0.006, 0.034) | 0.011 (-0.008, 0.029) | 0.222 (0.181, 0.263) |
|  |  | Happiness | 11,634 | 0.02 (-0.007, 0.047) | 0.012 (-0.011, 0.036) | 0.196 (0.152, 0.24) |
|  |  | Sleep Quality | 12,737 | -0.002 (-0.026, 0.022) | -0.001 (-0.021, 0.019) | 0.082 (0.038, 0.126) |
|  |  | Stressors | 12,737 | 0.003 (-0.022, 0.028) | 0.009 (-0.012, 0.029) | -0.131 (-0.178, -0.084) |
|  | Social Experiences | Loneliness | 12,735 | -0.011 (-0.035, 0.013) | 0.001 (-0.019, 0.02) | -0.143 (-0.187, -0.099) |
|  |  | Been outside | 12,659 | 0.002 (-0.016, 0.02) | 0.007 (-0.013, 0.027) | 0.001 (-0.046, 0.048) |
|  |  | Face-to-face | 12,147 | -0.003 (-0.022, 0.015) | -0.015 (-0.041, 0.011) | 0.103 (0.053, 0.153) |
|  |  | Telephone call | 12,325 | 0 (-0.018, 0.018) | -0.002 (-0.021, 0.016) | 0.03 (-0.017, 0.077) |
|  | COVID-19 Awareness | Knowledge | 12,737 | 0.004 (-0.017, 0.024) | 0.019 (-0.001, 0.039) | 0.302 (0.255, 0.349) |
|  |  | Information seeking | 12,731 | 0.004 (-0.015, 0.023) | 0.006 (-0.015, 0.026) | 0.127 (0.077, 0.177) |

**Table S8:** Model Results, fixed effects models.

|  | Separate | | Simultaneous | |
| --- | --- | --- | --- | --- |
| Variable | N | β (95% CI) | N | β(95% CI) |
| Remote working | 51,581 | 0.018 (0.007, 0.028) | 51,437 | 0.017 (0.006, 0.029) |
| Work outside house | 51,582 | -0.018 (-0.028, -0.009) | 51,437 | -0.016 (-0.026, -0.007) |
| Caring for adult | 51,587 | -0.009 (-0.017, 0) | 51,437 | -0.007 (-0.016, 0.002) |
| Volunteering | 51,584 | -0.005 (-0.017, 0.007) | 51,437 | -0.002 (-0.013, 0.009) |
| Chores | 51,586 | 0.012 (0.003, 0.021) | 51,437 | 0.009 (-0.001, 0.018) |
| Caring for children | 51,584 | -0.002 (-0.014, 0.01) | 51,437 | -0.003 (-0.015, 0.009) |
| Exercise | 51,575 | -0.009 (-0.02, 0.002) | 51,437 | -0.011 (-0.022, 0) |
| Community group | 51,586 | -0.016 (-0.031, -0.001) | 51,437 | -0.013 (-0.028, 0.001) |
| Arts and Crafts | 51,578 | 0.01 (0.001, 0.018) | 51,437 | 0.009 (0, 0.018) |
| Broader leisure | 51,572 | 0.016 (0.007, 0.026) | 51,437 | 0.016 (0.006, 0.026) |

**Table S9:** Difference in standardized female and male coefficients, RI-CLPM models.

|  | Variable | X ➔ Compliance | Compliance ➔ X |
| --- | --- | --- | --- |
| Confidence in Institutions | Government | 0.004 (-0.007, 0.016) | 0 (-0.009, 0.008) |
|  | Health system | -0.011 (-0.022, 0.001)* | -0.009 (-0.019, 0.002) |
|  | Acquiring essentials | -0.004 (-0.014, 0.007) | -0.004 (-0.014, 0.006) |
| Mental Wellbeing | Depression | 0.02 (0.008, 0.032)* | 0.021 (0.01, 0.032)* |
|  | Anxiety | 0.006 (-0.007, 0.019) | 0.01 (0, 0.02)* |
|  | Worthwhileness | -0.018 (-0.026, -0.009)* | -0.031 (-0.041, -0.02)* |
|  | Happiness | -0.032 (-0.044, -0.021)* | -0.038 (-0.05, -0.025)* |
|  | Sleep Quality | -0.002 (-0.012, 0.009) | -0.006 (-0.016, 0.004) |
|  | Stressors | 0.002 (-0.008, 0.012) | 0.003 (-0.006, 0.013) |
| Social Experiences | Loneliness | 0.021 (0.011, 0.03)* | 0.024 (0.014, 0.033)* |
|  | Been outside | -0.011 (-0.02, -0.002)* | -0.009 (-0.018, 0.001)* |
|  | Face-to-face | -0.005 (-0.015, 0.005) | -0.006 (-0.018, 0.006) |
|  | Telephone call | -0.002 (-0.011, 0.007) | -0.002 (-0.012, 0.007) |
| COVID-19 Awareness | Knowledge | 0.014 (0.004, 0.024)* | 0.021 (0.011, 0.031)* |
|  | Information seeking | -0.005 (-0.014, 0.004) | -0.009 (-0.018, 0)* |
| * p < 0.05.  Confidence intervals and p-values calculated by taking difference in coefficients and pooling standard errors with following formula:  ${SE}_{pool}=\sqrt{\frac{(n_{male}-1)\cdot{SE}_{male}^{2}+(n_{female}-1)\cdot{SE}_{female}^{2}}{n_{male}+n_{female} - 2}}$ | | | |

## FIGURES


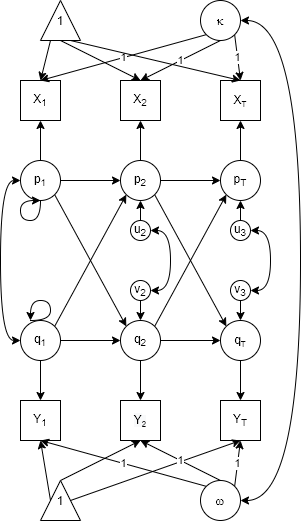


**Figure S1**: Hamaker et al. ^5^ RI-CLPM model (with means) for three-period case. Latent factors, κ and ɷ, are random intercepts. pt and qt are residuals in observed values, xt and yt, once time- and person-specific means are removed. Autoregressive and cross-lagged effects are modelled between these residuals.


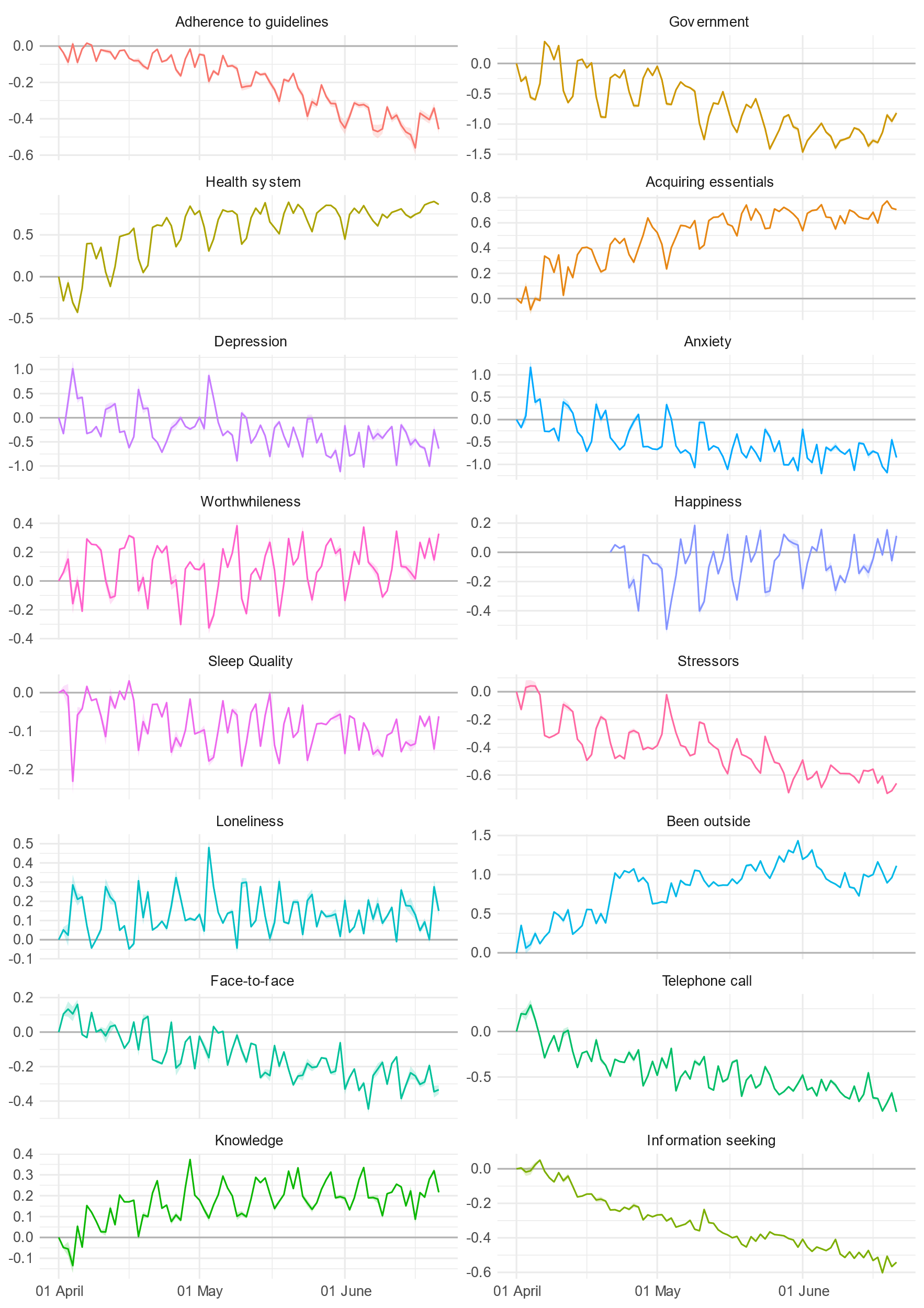


**Figure S2:** Time trends in self-reported compliance and non-time use variables. (Weighted) average daily values (+ 95% CIs) from sample with 11+ interviews between 01 April – 22 June. Scores are relative to average value on 01 April 2020. Note, y-axes on different scales.


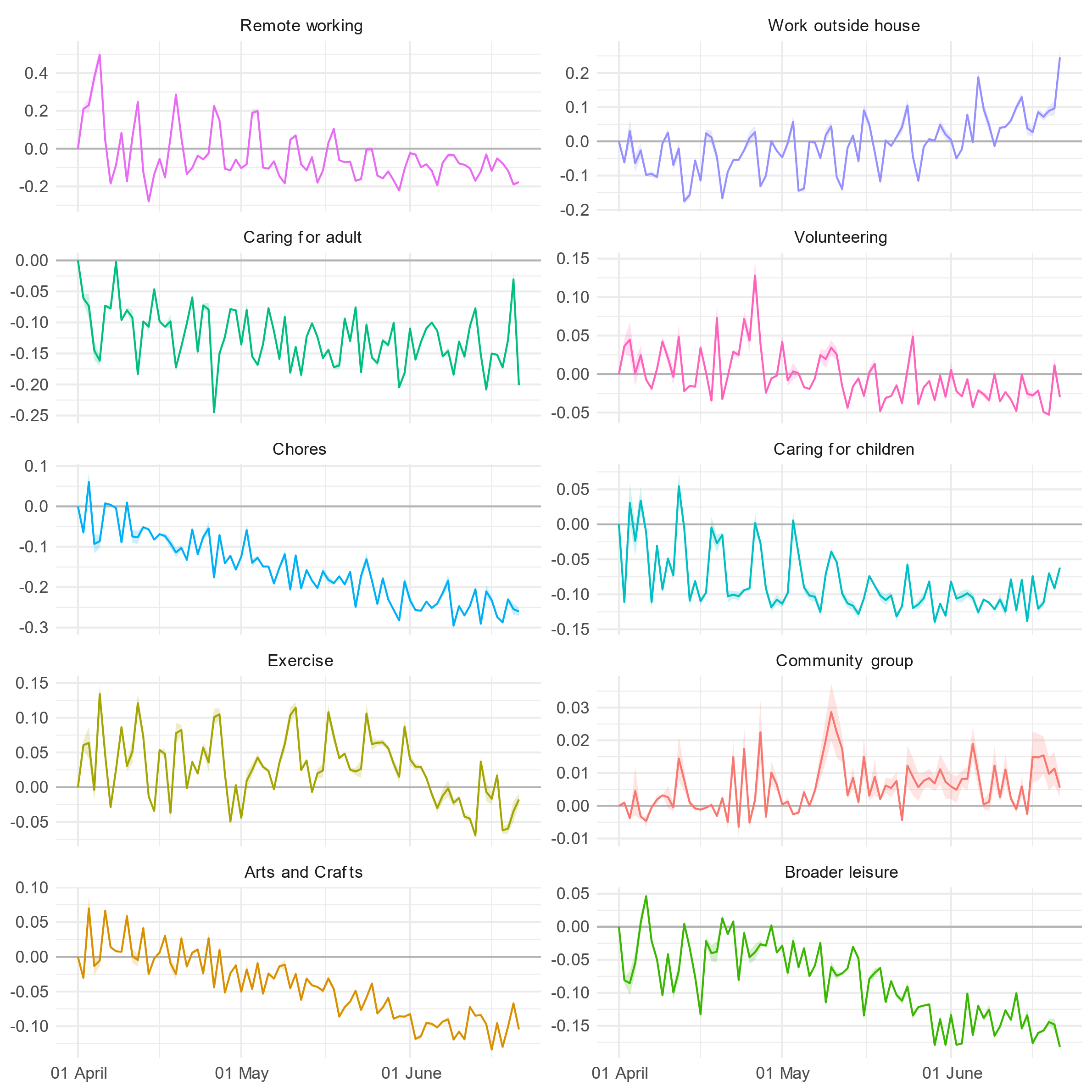


**Figure S3:** Time trends in time use variables. (Weighted) average daily values (+ 95% CIs) from sample with 11+ interviews between 01 April – 22 June. Scores are relative to average value on 01 April 2020. Note, y-axes on different scales.


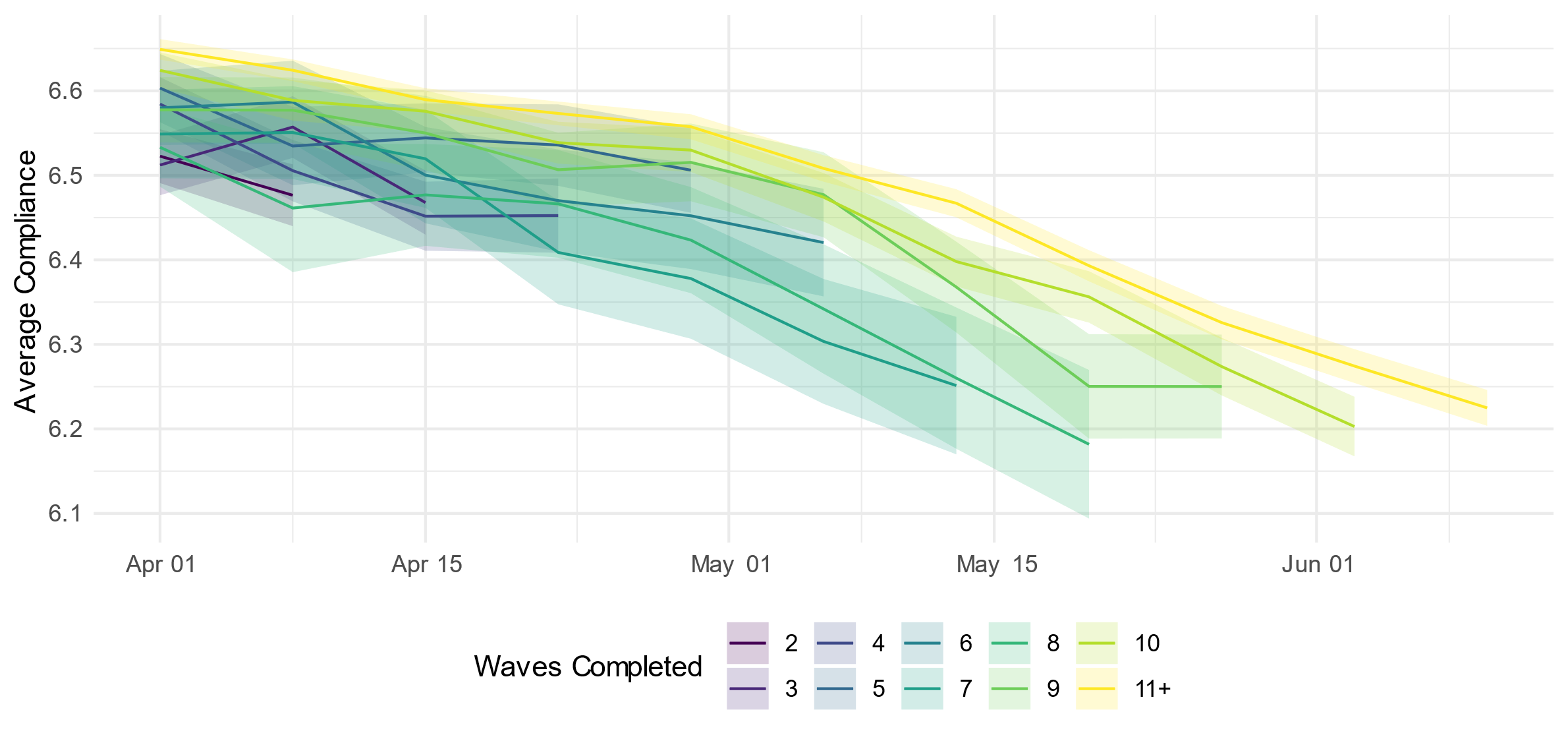


**Figure S4:** (Weighted) weekly average compliance levels by number of waves completed, among sample who were interviewed between 01 April – 07 April.


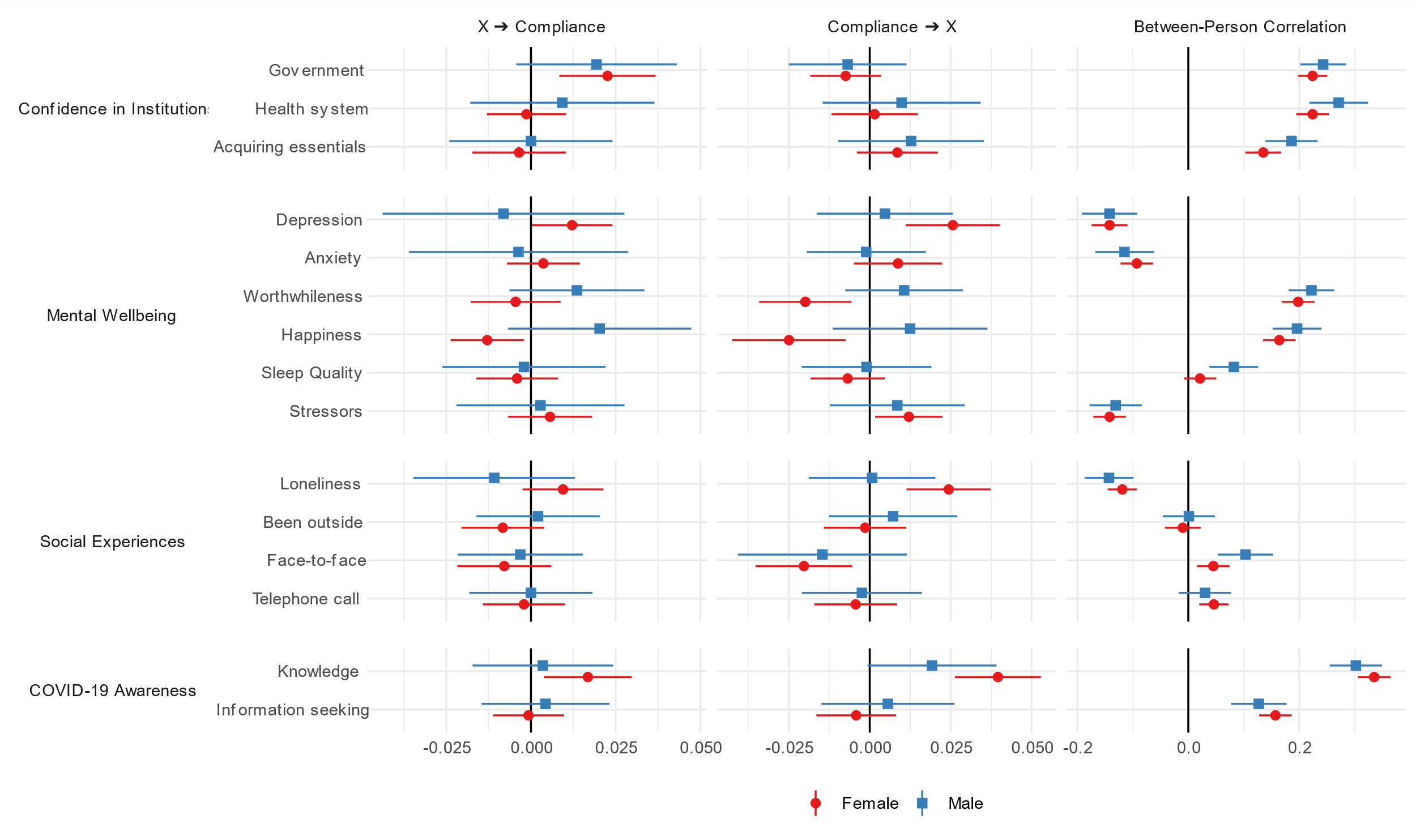


**Figure S5:** RI-CLPM Model Results stratified by gender. Left panel shows the cross-lagged effect of the exposure variable on compliance; the middle panel shows the cross-lagged of compliance on the exposure variable; and the right panel shows the correlation between the random intercept terms for the exposure and compliance


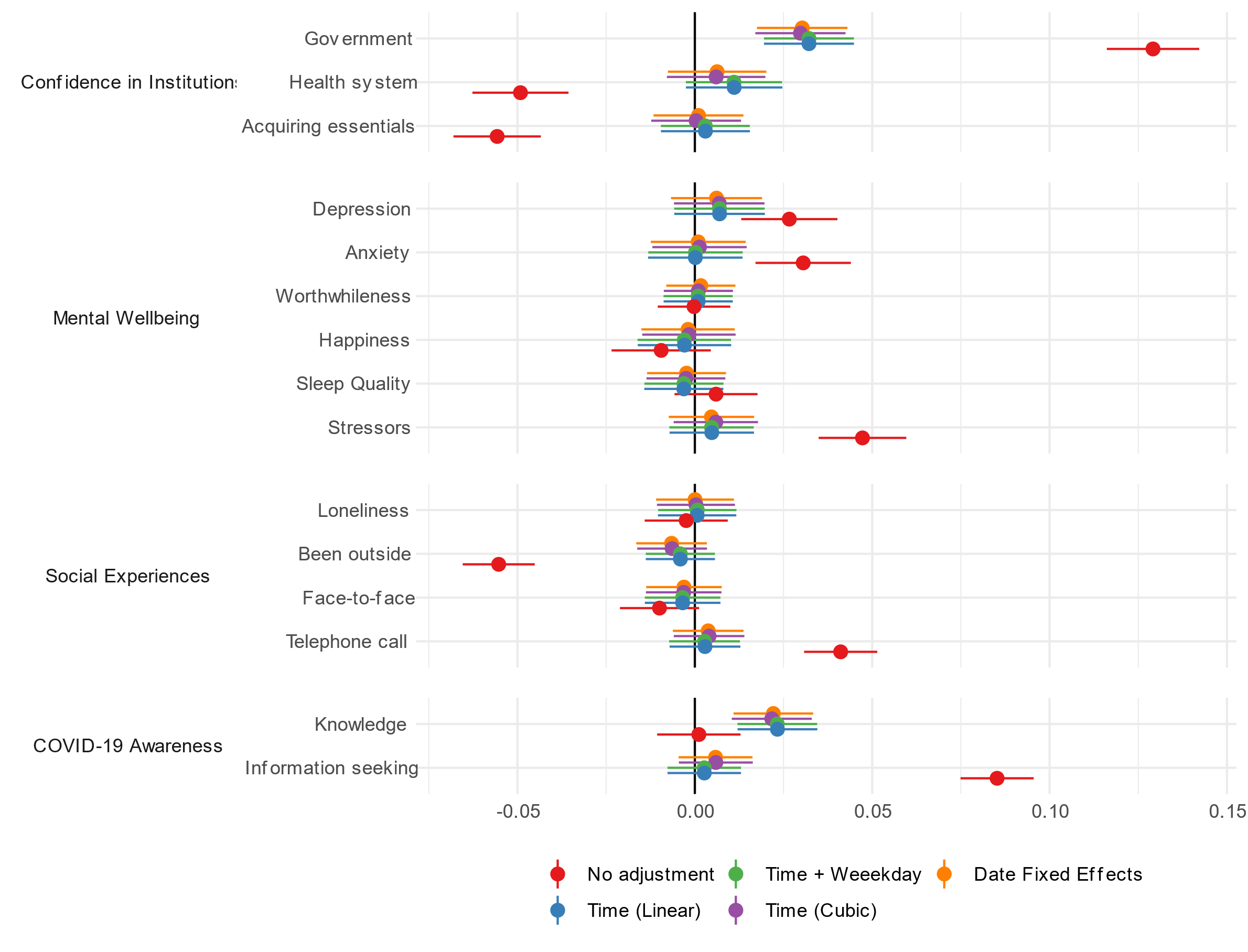


**Figure S6:** Results of fixed effects models with various methods for adjusting for time trends.


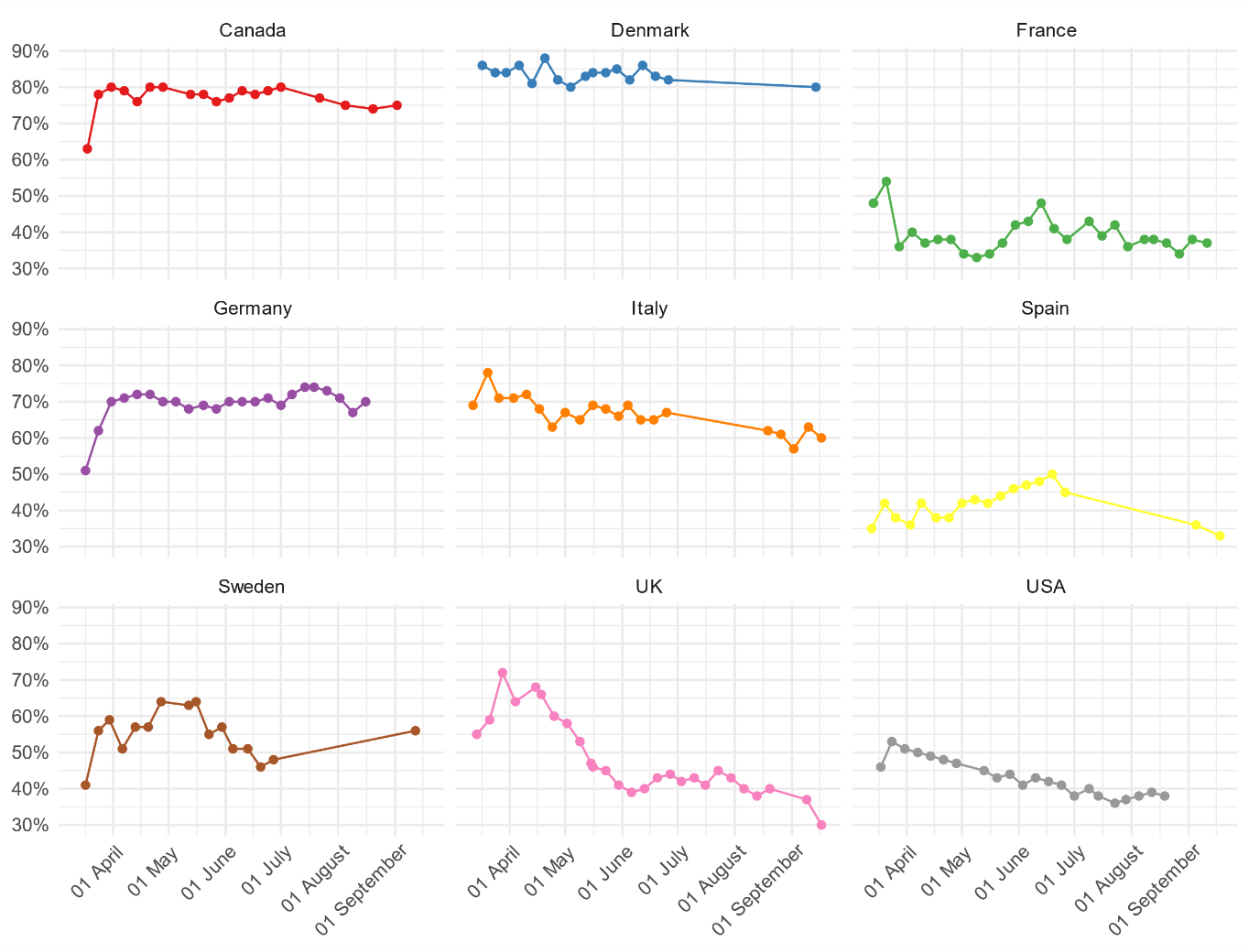


**Figure S7:** Proportion of people who think their government is handling the issue of coronavirus “very” or “somewhat” well. Source: YouGov ^6^


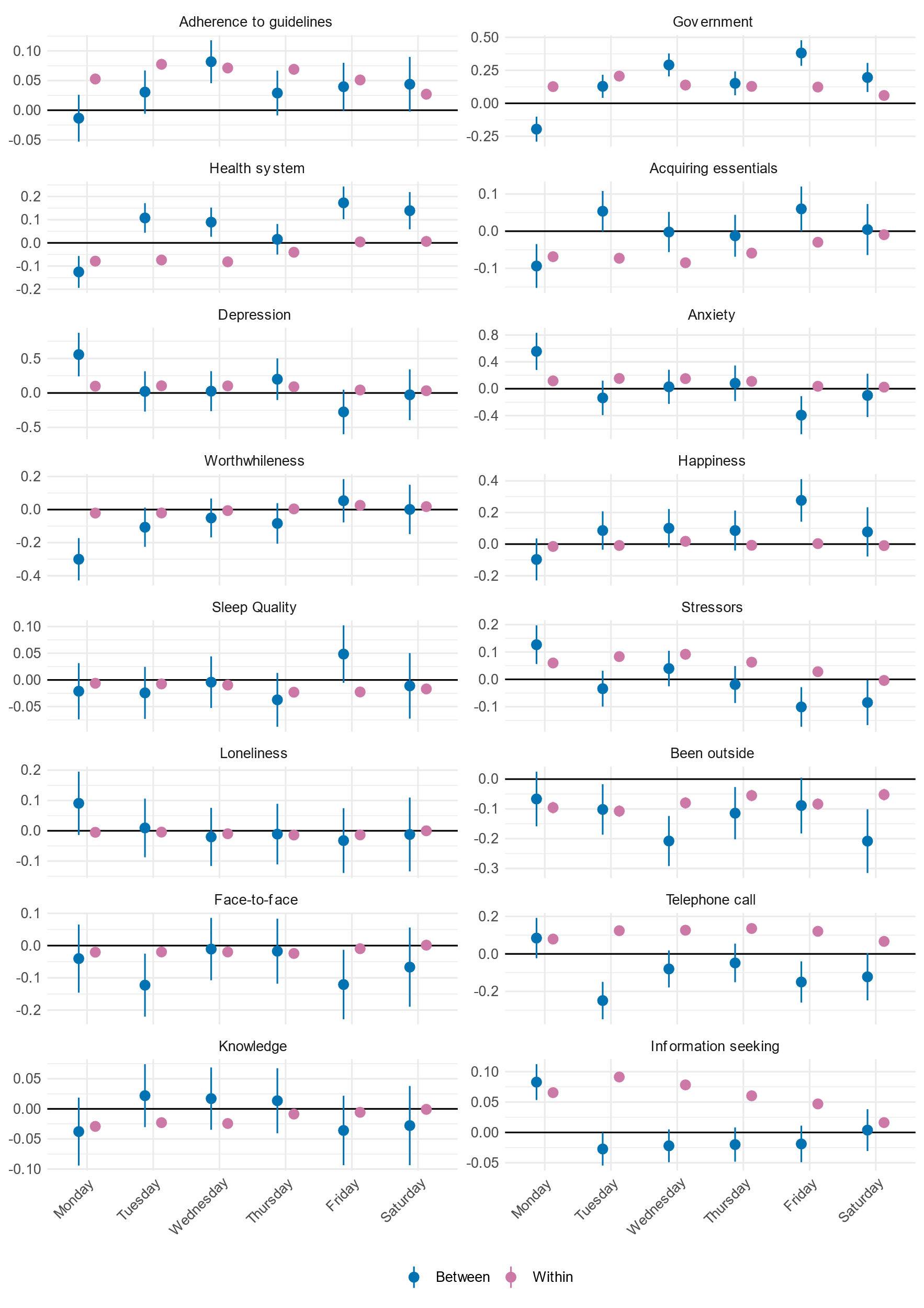


**Figure S8:** Within-person and between-person variation in self-reported compliance and non-time use variables by day of week. Derived from Random Effects Within-Between models. Note, reference category is Sunday and y-axes on different scales.


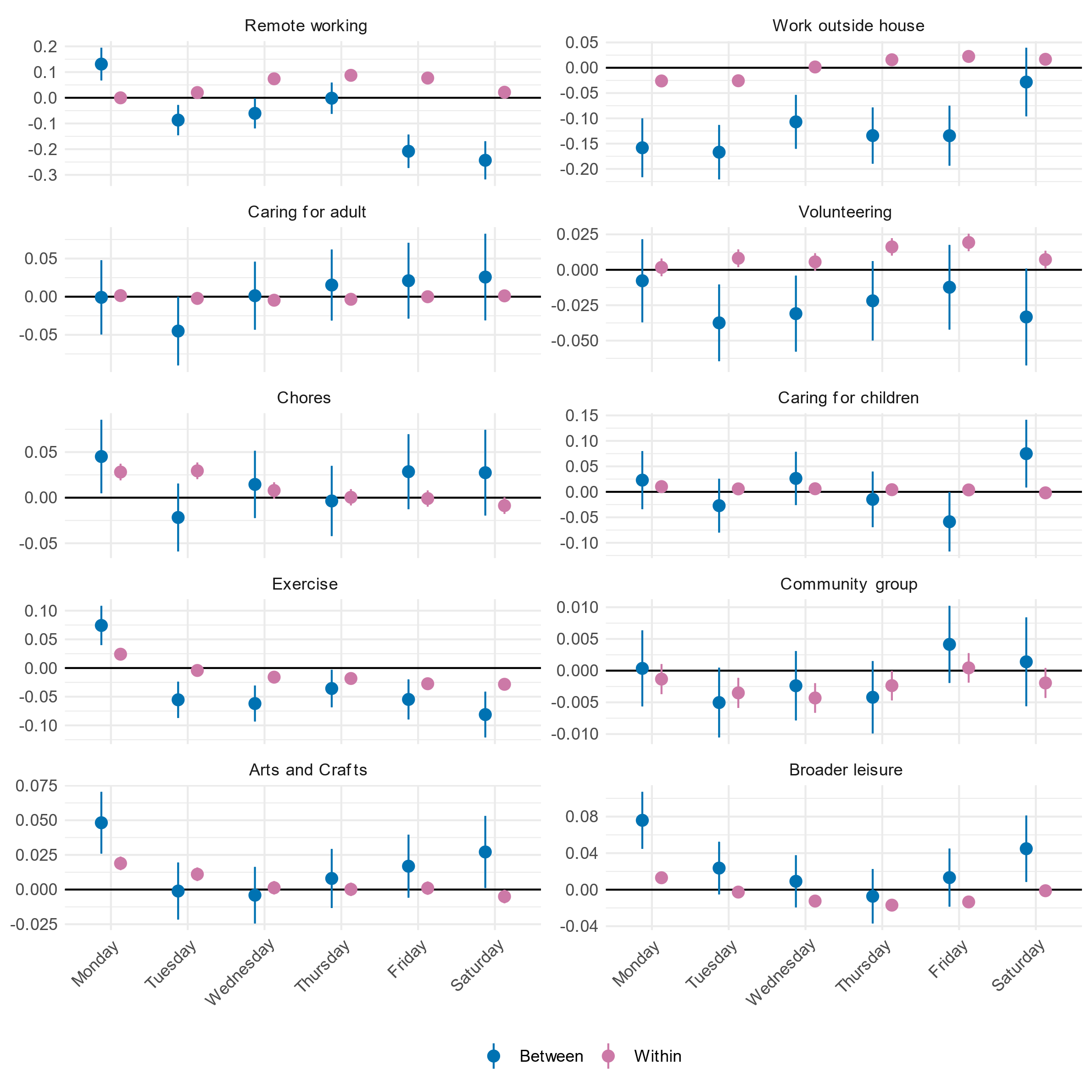


**Figure S9:** Within-person and between-person variation in time use variables by day of week. Derived from Random Effects Within-Between models. Note, reference category is Sunday and y-axes on different scales.


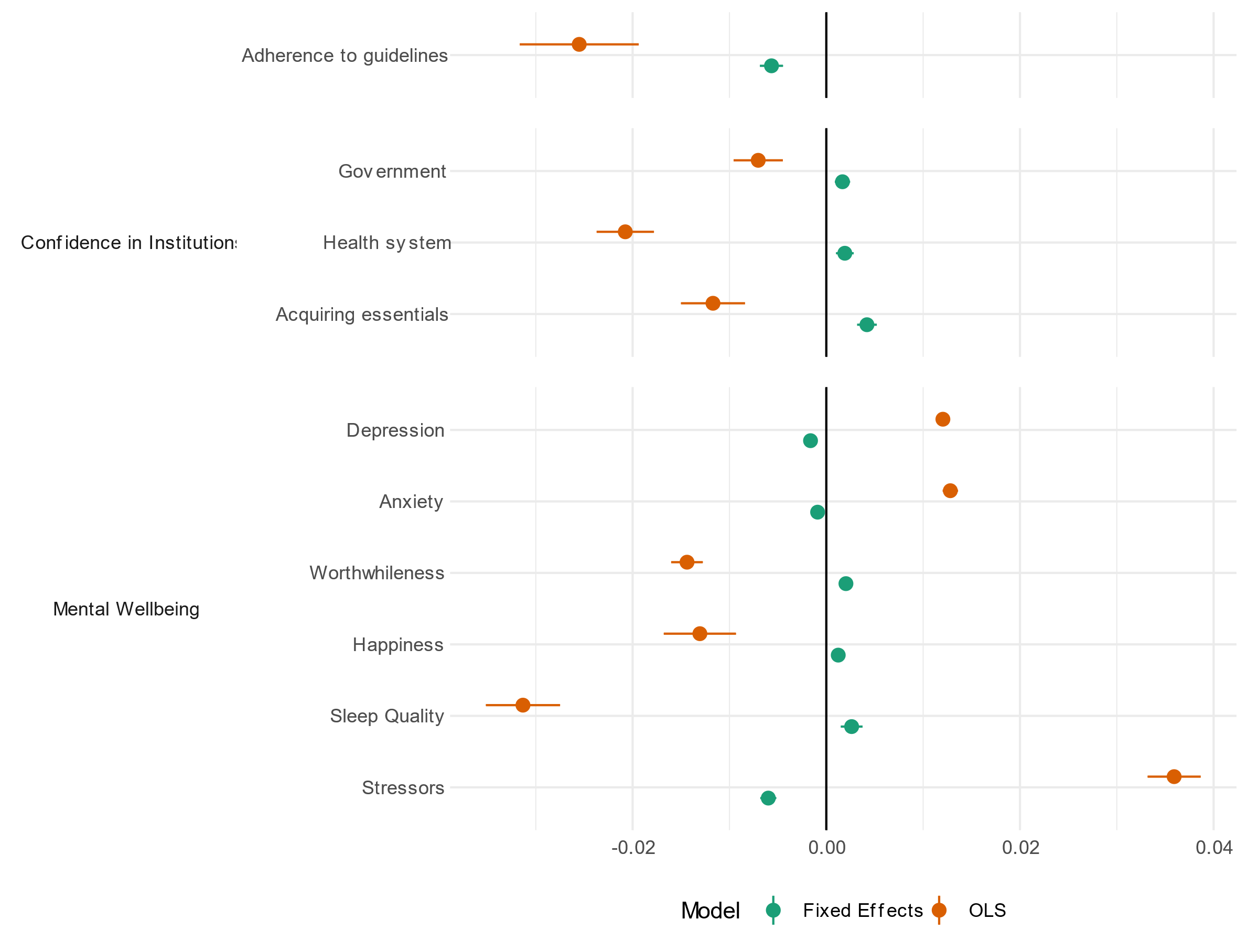


**Figure S10:** Analysis of attrition. OLS estimates refer to linear bivariate OLS regressions of attrition at any point from the survey and participant characteristics at first interview. Fixed effects estimates refer to fixed effects regression of attrition directly following interview and participant characteristics in interview. Models estimate for each characteristic separately and include cubic time trends to account for changes in drop-out through time.

**
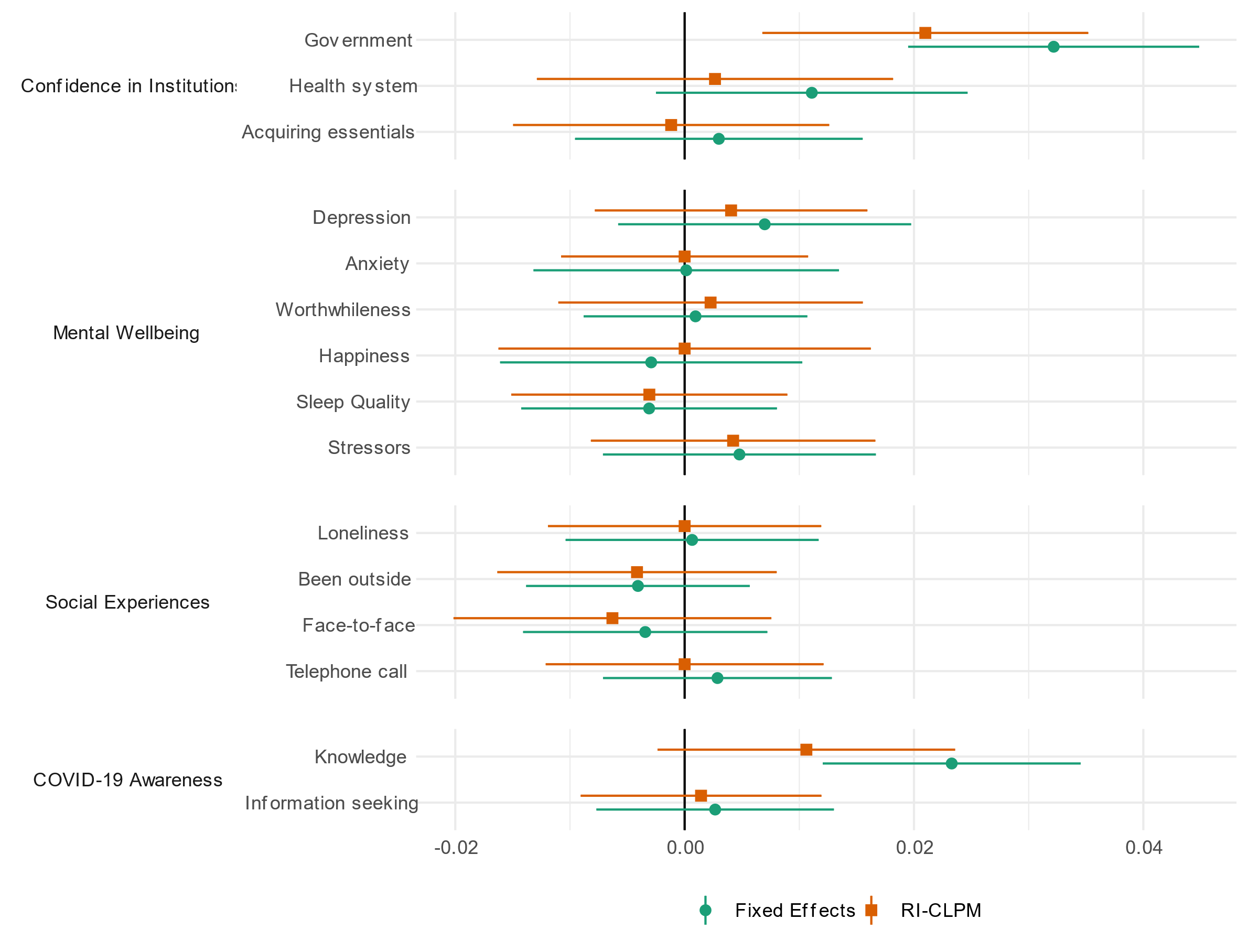
**

**Figure S11:** Comparison of results from RI-CLPM and fixed effects models (+95% CIs). Association between within-person change in measure and compliance at following wave, adjusting for linear time trends.

# References

1 Office for National Statistics. Personal well-being in the UK. 2020; published online July 30. https://www.ons.gov.uk/peoplepopulationandcommunity/wellbeing/bulletins/measuringnationalwellbeing/april2019tomarch2020 (accessed Sept 1, 2020).

2 Bell A, Fairbrother M, Jones K. Fixed and random effects models: making an informed choice. *Qual Quant* 2019; **53**: 1051–74.

3 Rosseel Y. lavaan : An R Package for Structural Equation Modeling. *J Stat Soft* 2012; **48**. DOI:10.18637/jss.v048.i02.

4 Nomis. Annual Population Survey data. 2018 https://www.nomisweb.co.uk/ (accessed Sept 28, 2018).

5 Hamaker EL, Kuiper RM, Grasman RPPP. A critique of the cross-lagged panel model. *Psychological Methods* 2015; **20**: 102–16.

6 YouGov. COVID-19: government handling and confidence in health authorities. 2020; published online Sept 18. https://yougov.co.uk/topics/international/articles-reports/2020/03/17/perception-government-handling-covid-19 (accessed Sept 21, 2020).
